# Tailoring Uptake Efficacy of HSV-1 gD Tailoring Uptake Efficacy of Hsv-1 GD Derived Carrier Peptides

**DOI:** 10.3390/biom10050721

**Published:** 2020-05-06

**Authors:** Szilvia Bősze, Ferenc Zsila, Beáta Biri-Kovács, Bálint Szeder, Zsuzsa Majer, Ferenc Hudecz, Katalin Uray

**Affiliations:** 1 MTA-ELTE Research Group of Peptide Chemistry, 112, P.O. Box 32, H-1518 Budapest, Hungary; bosze@elte.hu (S.B.); beabiri@caesar.elte.hu (B.B.-K.); fhudecz@elte.hu (F.H.); 2 Institute of Materials and Environmental Chemistry, Research Centre for Natural Sciences, P.O. Box 286, H-1519 Budapest, Hungary; zsila.ferenc@ttk.mta.hu; 3 Eötvös Loránd University, Institute of Chemistry, Budapest, 112, P.O. Box 32, H-1518 Hungary; majer@chem.elte.hu; 4 Institute of Enzymology, Research Centre for Natural Sciences, P.O. Box 286, H-1519 Budapest, Hungary; szederbalint@gmail.com

**Keywords:** carrier peptide, HSV-1 gD glycoprotein, Nectin-1, Herpesvirus entry mediator, cellular uptake, intracellular localisation, helical propensity, structure – activity studies

## Abstract

Regions of the *Herpes simplex* virus-1 (HSV-1) glycoprotein D (gD) were chosen to design carrier peptides based on the known tertiary structure of the virus entry receptor complexes. These complexes consist of the following: HSV-1 gD–nectin-1 and HSV-1 gD–herpesvirus entry mediator (HVEM). Three sets of peptides were synthesised with sequences covering the (i) *N*-terminal HVEM- and nectin-1 binding region -5–42, (ii) the 181–216 medium region containing nectin-1 binding sequences and (iii) the *C*-terminal nectin-1 binding region 214–255. The carrier candidates were prepared with acetylated and 5(6)-carboxyfluorescein labelled *N*-termini. The peptides were chemically characterised and their conformational features in solution were also determined. In vitro internalisation profile and intracellular localisation were evaluated on SH-SY5Y neuroblastoma cells. Peptide originated from the *C*-terminal region 224–247 of the HSV-1 gD showed remarkable internalisation compared to the other peptides with low to moderate entry. Electronic circular dichroism secondary structure studies of the peptides revealed that the most effectively internalised peptides exhibit high helical propensity at increasing TFE concentrations. We proved that oligopeptides derived from the nectin-1 binding region are promising candidates—with possibility of Lys^237^Arg and/or Trp^241^Phe substitutions—for side-reaction free conjugation of bioactive compounds—drugs or gene therapy agents—as cargos.

## 1. Introduction

Applications of virus-derived peptides have been described in various fields such as targeted drug and gene transport strategies, development of vaccines, diagnostics and imaging techniques [1,2,3]. Viruses considered to be pathogenic to humans and other organisms can be promising tools due to their unique characteristics of mastering the ability to enter different target cells efficiently and to overcome all the barriers present both in the cells and the host organism. The virus-derived carrier peptides are suitable multifunctional vehicles for the transportation of various compounds to target cells.

The *Herpesviridae* family consists of various viruses: among the human herpes viruses (HHV) the *Herpes simplex* virus (HSV) 1 and 2, and the *Varicella zoster* virus (VZV) belong to the α-*Herpesvirinae*; cytomegalovirus (CMV), HHV 6A, 6B and 7 to β-*Herpesvirinae*; rhadinoviruses like Epstein-Barr virus (EBV) and Kaposi’s sarcoma associated virus to the γ-*Herpesvirinae*. HSV-1 infection causes cold sores and ulcers on the face. These symptoms, in most cases, clear with the help of presently available topical treatment, but the virus becomes latent in the cells of peripheral nervous system and is frequently reactivated. During reactivation, the virus is transported via the axons of the neurons to the skin causing new sores. In certain cases, serious complications may arise affecting the nervous system (encephalitis, meningitis).

The *Herpesviridae* have relatively large (120–200 kb), double-stranded linear DNA genome within an icosahedral capsid, which is in a lipid bilayer envelope covered with membrane bound glycoproteins [4]. Five HSV envelope glycoproteins are participating in the *Herpes simplex* virus entry, gC, gB, gD and the heterodimer gH/gL. Among these the role of gD glycoprotein is unique among the *Herpes* viruses [5]. The ectodomain of HSV-1 gD glycoprotein contains a compact Ig-like β-sandwich domain (residues 56 – 184) which is wrapped by α-helices, strands and several loops consisting of the *N*-terminal (residues P^23^ to ^I55^) and *C*-terminal (residues A^185^ to E^259^) extensions [6], the 1–22 region was not crystallisable.

During virus entry, after tethering the virus by nonspecific interactions of gC and gB to the host cell surface, interaction between the receptor-binding protein gD and a specific cell surface receptor (herpesvirus entry modulator A (HVeA or HVEM) or nectin-1) follows. During the binding process, the structure of gD undergoes changes, the 50 residues long *C*-terminal pro-fusion domain (residues T^260^ to A^310^), previously wrapped on the protein [7,8], unfolds, and nectin-1 [6,9] or HVEM [10] binds to the new surface left exposed by this domain. The receptor binding by gD follows gD’s binding to gB [11], triggering the membrane fusion process that is mediated by gB and gH/gL [5,12].

**Figure 1:**
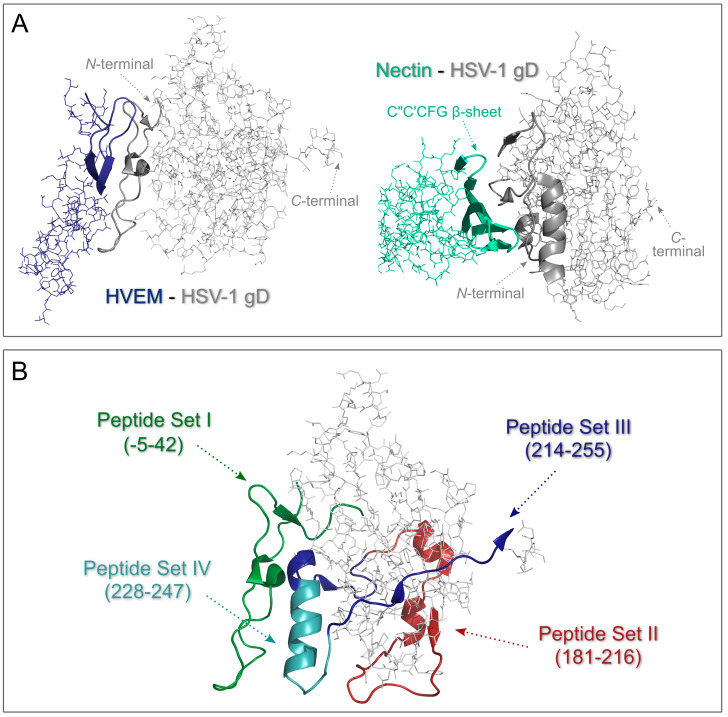
(**A**) Structure of HVEM – HSV-1 gD complex (1JMA) and the first domain of nectin-1 in complex with HSV-1 gD (3U82) with the binding interface shown in ribbon model. Similar orientation of the HSV-1 gD protein has been intended, but the conformation of the protein is not identical in the different complexes. (**B**) Localisation of the regions defining the four peptide sets (I-IV) designed for synthesis based on the sequence of HSV-1 gD glycoprotein (PDB ID: 1JMA).

Recombinant HSV-1 gD 1-285 protein was co-crystallised with recombinant nectin-1 [6,9] and HVEM [10] proteins. According to Di Giovine et al. (3SKU) [9] and Zhang et al. (3U82) [6], the nectin-1 binding sites of HSV-1 gD are mainly located in the terminal extensions, with only a few residues of the Ig-like domain and two short regions near the N-terminus making contact with nectin-1. From the part of nectin-1 the C”C’CFG β-sheet is the main participant in this contact. In the study of Carfi et al. (1JMA) [10], it is shown that HVEM is bound to parts of the *N*-terminal 1-37 hairpin structure of HSV-1 gD. The binding interface of HSV-1 gD with HVEM (1JMA) and the first domain of nectin-1 in complex with HSV-1 gD (3U82) is shown on Figure 1A.

The importance of gD glycoprotein has been demonstrated by Cocchi et al. in 2004 [13] by gD-null mutant non-infecting virus. Infectivity was restored by adding to the gD-null mutant viruses either the soluble ectodomain of gD or mutant virus-bound gDs containing both the receptor binding region and the profusion domain.

Glycoprotein gD also plays an important role in cell-to-cell spread of the infection, Krummenacher et al. has shown that newly expressed gD accumulates on the surface of infected cells near neighbouring, uninfected cells bearing nectin-1 [14].

The gH glycoprotein has been extensively studied for effective targeting peptides, and the 20mer gH ^625^HGLASTLTRWAHYNALIRAF^644^ forming an amphiphilic helix when in connection with lipid membranes, is able to non-specifically deliver cargo molecules, even proteins, across cell membranes more efficiently than polycationic cell penetrating peptides (e.g., Tat) [15]. The same peptide used in the form of dendrimers proved that the cellular entry is based on pore formation, avoiding endocytosis [16].

In this study, our aim was to design carrier peptides based on the entry process of HSV-1 involving gD glycoprotein – HVEM – nectin-1 complex. The sequences of the peptides were derived from the gD glycoprotein of HSV-1, by using sets of overlapping sequences based on the structure and interaction of the entry complex’ proteins described in the studies above [6,9,10]. The designed sequences of the peptides are mainly based on the regions originated from HSV-1 gD in close proximity of either nectin-1 or HVEM (Figure 1B). We postulated that certain peptides possess the ability to internalise into cells and can also be used as delivery vehicles.

## 2. Materials and Methods

### 2.1. Materials

9-Fluorenylmethoxycarbonyl (Fmoc) protected amino acid derivatives and Rink amide 4-methylbenzhydrylamine (Rink amide MBHA) resin were purchased from IRIS Biotech GmbH (Marktredwitz, Germany). The reagents for synthesis were *N,N’*-diisopropylcabodiimide (DIC), *N,N*-diisopropyl-ethylamine (DIEA), 1-hydroxybenzotriazole (HOBt), 1,8-diazabicyclo-[5.4.0]undec-7-ene (DBU), piperidine, thioanisol, phenol, 1,2-ethanedithiol, 5(6)-carboxyfluorescein (Cf), and they were purchased from Sigma-Aldrich (Budapest, Hungary). Trifluoroacetic acid (TFA) and solvents (*N,N*-dimethylformamide (DMF) and dichloromethane) (DCM)) for synthesis, as well as HPLC grade acetonitrile, were obtained from Molar Chemicals (Halásztelek, Hungary). Cf-Penetratin was provided by Kata Horváti (MTA-ELTE Research Group of Peptide Chemistry; Eötvös Loránd University, Institute of Chemistry) [17,18].

### 2.2. Peptide Synthesis

Eleven 20mer peptides derived from the HSV-1 gD (from HSV-1 gD regions -5–23, 181–216 and 228–255) were prepared by multipin solid phase synthesis method on Rink amide linker functionalised Mimotopes cleavable lanterns (Mimotopes, Clayton, Australia) [19] with Fmoc/tBu strategy on 8 micromolar scale, in duplicates. After the completion of the synthesis, one copy of all the peptides was labelled with 5(6)-carboxyfluorescein (Cf) on their *N*-termini, the other copy was acetylated, and then the peptides were cleaved from the lanterns by TFA – thioanisol – water – phenol – 1,2-ethanedithiol (80:5:5:7.5:2.5, *v/v/v/m/v*) cleavage mixture removing the side chain protecting groups as well, except the acetamidomethyl groups from Cys residues of Set II peptides. The rest of the peptides were prepared on Rink-amide MBHA resin, partly on a Syro-I automated peptide synthesiser (Biotage, Uppsala, Sweden); Cf-(228–243) and its Lys → Arg / Trp → Phe modified versions, the truncated Cf-(228–243 F) and the Cf-(10–33) peptide and its *N*- and *C*-terminal decamer fragments were prepared manually with resin portioning on a 0.05 millimolar scale. Acetylated and Cf-labelled versions were prepared, and the peptides were cleaved from the resin with the above cleavage mixture. In case of sequences 214–233 and 219–238 the methionine residue has been substituted by norleucine. The peptides were purified by RP-HPLC on a Knauer HPLC system (Berlin, Germany) on Phenomenex Jupiter C18 column (10 μm, 300 Å, 10 mm × 250 mm). The purity and identity of the products were studied with RP-HPLC and ESI-MS.

### 2.3. Stability Studies of Synthetic Peptides

Before the cellular uptake studies the stability of the Cf-labelled synthetic peptides was determined. Peptides were dissolved at 1 mg/mL concentration in serum-free DMEM medium (Lonza, Basel, Switzerland), and incubated at 37 °C for 24 h. Samples for RP-HPLC (Knauer, Berlin, Germany) were taken at 0, 3, 6 and 24 h. Phenomenex Luna C18 column (5 μm, 300 Å, 4.6 mm × 250 mm) (Gen-Lab Ltd., Budapest, Hungary) was applied with a gradient of 0–5 min 5% B eluent elevated to 55% B during 25 min, where eluent A: 0.1% TFA/water, eluent B: 0.1% TFA/acetonitrile–water 80:20 (*v/v*).

### 2.4. Fluorescence Spectroscopy of the 5(6)-Carboxyfluorescein Labelled Peptide Derivatives

The fluorescence properties of the Cf-peptides were studied at different pH values with fluorescence spectroscopy using Varian Cary Eclipse fluorimeter (the method was first described in [20]). Briefly: peptides were dissolved in 0.1 M citrate phosphate buffer (pH = 4.0, 5.0, 6.0, 7.0); and 0.4 μm peptide concentration was used. Excitation wavelength was λ = 488 nm (corresponding to the laser of the flow cytometer). Detection was in the range of 500–700 nm.

### 2.5. Secondary Structure Prediction

For the 3D visualisation of the secondary structure of HSV-1 gD peptides PEP-FOLD3 prediction was used [21], number of simulations: 100, sorted by sOPEP score [22].

### 2.6. Electronic Circular Dichroism (ECD) Spectroscopy

ECD spectra of acetylated HSV peptides were recorded using a Jasco J-810 and Jasco J-715 spectropolarimeter (Jasco Corporation, Tokyo, Japan) in the λ = 180–270 nm wavelength range using a 0.02 or a 0.1 cm path length quartz cell at room temperature, under constant nitrogen flush with continuous scanning mode. All spectra reported here were obtained as an average of three or five individual scans and corrected with the solvent ECD spectra. The J-810 equipment was calibrated with ammonium α-10-camphor-sulfonate. The samples were dissolved in trifluoroethanol (TFE) (Sigma), distilled water and a 1:1 (*v/v*) mixture of TFE:water. The solution concentration was in the range of 0.5–0.7 mg/mL (J-810) or ~0.14 mg/mL (J-715) for peptide amides. ECD band intensities are expressed in mean residual molar ellipticity unit. The percentage of the different conformational elements contributing to the ECD spectra was calculated by the MS Excel version of the PEPFIT program developed originally by Reed and Reed [23,24]. The algorithm estimates the percentage of secondary contents by fitting experimental ECD data to the combination of reference spectra. The best fit is defined by the R^2^ value, where an R^2^ = 1 corresponds to a perfect match.

### 2.7. Cell Culturing and Cellular Uptake Studies

The SH-SY5Y cells were kindly provided by Dr. Zsolt Datki (Department of Medical Chemistry, University of Szeged) [25,26,27,28] and maintained as adherent cultures in DMEM (Lonza™) supplemented with 10% heat-inactivated fetal calf serum (FCS, Lonza™), L-glutamine (2 mM), 1% non-essential amino acids (Sigma), 1 mM sodium pyruvate (Sigma), and penicillin-streptomycin antibiotics mixture (Lonza™ BioWhittaker™, 5000 IU/5000 IU). Cells were grown in sterile T25 or T75 flasks with ventilation cap (Sarstedt, Nümbrecht, Germany) at 37 °C in a humidified atmosphere with 5% CO_2_. Prior to experiments SH-SY5Y cells were seeded into 24-well plates (Sarstedt) in DMEM medium (supplemented as described above), 5 × 10^4^ cells/well in 1 mL and cultured overnight at 37 °C, 5% CO_2_. After centrifugation for 5 min at 1000 rpm the medium was removed, 5(6)-carboxyfluorescein labelled peptides have been added in serum-free DMEM at the concentration range of 0.4 – 50 or 250 μM in 400 μL/well, and incubated for 3 h (37 °C, 5% CO_2_). Then the cells were washed twice with serum-free DMEM. After the washing steps all supernatant was removed, and 100 μL 0.25% trypsin (Sigma-Aldrich) was added to the cells. After 5 min incubation, 0.8 mL 10% FCS/HPMI medium was added (HPMI buffer contained 9 mM glucose, 10 mM NaHCO_3_, 119 mM NaCl, 9 mM HEPES, 5 mM KCl, 0.85 mM MgCl_2_, 0.053 mM CaCl_2_, 5 mM Na_2_HPO_4_ × 2H_2_O, pH 7.4 [23]) and the cells were transferred into FACS tubes (Sarstedt), centrifuged again. After centrifugation cells were resuspended in 250 μL HPMI, and the cellular uptake of the peptides was analysed by measuring the intracellular fluorescence on a BD LSR II flow cytometer (excitation: λ = 488 nm (Coherent Sapphire laser), emission channel: LP510). The resulting intracellular fluorescence intensity data were evaluated by FACSDiva 5.0 software.

### 2.8. Intracellular Localisation Studies using Confocal Laser Scanning Microscopy

SH-SY5Y cells were seeded into coverslips (thickness 1, Assistent®, Karl Hecht GmbH & Co KG, Sondheim vor der Rhön, Germany) containing 24-well plates (Sarstedt) for microscopy studies at a density of 7.5 x 10^4^ cells/well, in 1 mL complete DMEM medium. The following day, cells were treated with Cf-HSV peptides at a concentration of 25 µM (diluted in serum free DMEM medium) for 3 h. Lysosomes were stained with Lysotracker Deep Red (Thermo Fisher Scientific, Waltham, MA, USA) according to the manufacturer’s instructions. Nuclei were visualised with Hoechst 33342 (Thermo Fisher Scientific, 0.2 µg/mL). After each step, cells were washed three times with serum-free medium. Following the staining and washing steps, cells were fixed with 4% paraformaldehyde (Sigma-Aldrich, Budapest, Hungary), the solution was prepared in-house, washed with PBS (pH = 7.4). The coverslips were mounted with Mowiol 4–88 (Sigma-Aldrich, Budapest, Hungary) to microscopy slides. Imaging was performed by a Zeiss LSM-710 system (Carl Zeiss microscopy GmbH, Oberkochen, Germany) with a 40×/1.4 Plan-Apochromat oil immersion objective using lasers with excitation maxima 405, 488 and 633 nm for detecting Hoechst, Cf-conjugated peptides and LysoTracker, respectively. Images were processed with ZEN (Carl Zeiss microscopy GmbH).

## 3. Results

### 3.1. Synthesis and Characterisation of HSV-1 gD Peptides

Peptides to be synthesised for ECD spectroscopic structural and cellular uptake studies were selected using the known X-ray structure of HSV-1 gD – nectin-1 complex (3U82) [6,9] and that of the HSV-1 gD – HVEM complex (1JMA) (Figure 1A) [10]. Peptide regions were selected based on the regions of HSV-1 gD contacting either nectin-1 or HVEM according to the above complex structures, elongated towards non-contacting regions. Thus, overlapping peptides from the HSV-1 gD -5–42, 181–216 and 214–255 regions were selected (Figure 1B).

Twenty-eight peptide sequences corresponding to the nectin-1/HVEM binding site of HSV-1 gD glycoprotein were synthesised on solid phase, either labelled with 5,6-carboxyfluorescein or acetylated, cleaved from the resin, purified by RP-HPLC and identified with ESI-MS (MS spectra of Cf-peptides are presented in the Supplementary information, Figures S1–S4). The acetamido protecting group has not been removed from peptides containing Cys residues to prevent oxidation and to mimic the disulphide bond within the protein. The peptides obtained were of more than 95% purity. The code, structure and analytical data of the Cf-labelled peptides are shown in Table 1. Peptides designed for secondary structure studies were acetylated, their analytical data are shown in Table S1 of Supplementary information. The numbering of the peptide sequences is according to the protein lacking the signal sequence; two peptides from the *N*-terminal of the HSV-1 gD contain 1–5 amino acids from the signal region have negative numbering.

Some of the Set I peptides contained methionine residue. Methionine in Set III peptides was replaced by norleucine, with identical side chain size, but the sulphur atom is replaced by a methylene group, because the methionine is sensitive for oxidation. In Set IV aromatic side chain of oxidatively sensitive tryptophan was changed by phenylalanine; and the basic lysine was replaced by the similar, but less reactive arginine.

Peptide amides prepared manually by multipin synthesis on Mimotopes lanterns (Figure S5) proved to be similar in quality as peptides prepared on Syro-I automated peptide synthesiser (Figure S6). The 8 µmolar scale of the crude Cf-labelled 20mer peptides was sufficient for purification, characterisation and cellular uptake studies; therefore, preliminary screening of even 96 peptides would be feasible with this method for laboratories without access to automated synthesiser. For further studies of lead peptides larger scale synthesis is practical. Total manual synthesis on resins is also useful for families of peptides (amino acid replacement, *N*-terminal truncation) with resin division (Figure S7).

**Figure 2:**
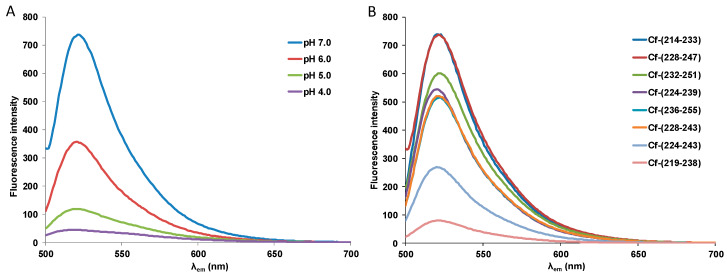
Fluorescence emission spectra of (**A**) peptide Cf-(228–247) at different pH values and of (**B**) Set III Cf-HSV peptides from the 214–255 region of HSV-1 gD (pH = 7.0).

The Cf-labelled HSV-1 gD peptides were characterised by their fluorescence spectra as well. Fluorescence intensity spectra of Set III peptides are shown in Figure 2. At pH = 4.0 all Cf-peptides showed an average of 93% decrease in fluorescence intensity compared with that measured at pH = 7.0, a characteristic feature of 5(6)-carboxyfluorescein. In case of lysosomal localisation of peptides, therefore, the intracellular fluorescence would be negligible. The fluorescence intensity in the case of most peptides at identical pH varied with cc. 1:2 ratio between the ones with the lowest and the highest fluorescence, with the exception of peptides Cf-(228–243 F) and Cf-(224–243) with lower fluorescence, and most notably Cf-(219–238). The latter showed only 13% fluorescence intensity compared to the average maximum intensity. Fluorescence intensity spectra (at pH = 4.0 – 7.0) of all peptides can be seen in the Supplementary information (Figures S8–S11). The position of the maximum emission wavelength did not vary more than 2 nm; therefore, the Cf-labelled peptides are suitable for comparative analysis in flow cytometry.

The stability of the Cf-labelled peptides was studied in serum-free DMEM medium for 0, 3, 6 and 24 h at 37 °C. Peptides containing methionine residues have been partly oxidised within 3 hours, and completely within 24 hours, other peptides proved to be stable under these circumstances, except for a slight decrease in the intensity of the peak corresponding to the intact peptide at 24 h. As an example, HPLC chromatograms of the peptides Cf-(228–247) and Cf-(236–255) are shown in the Supplementary information (Figure S12).

### 3.2. Secondary Structure Studies Using Electronic Circular Dichroism Spectroscopy

**Figure 3:**
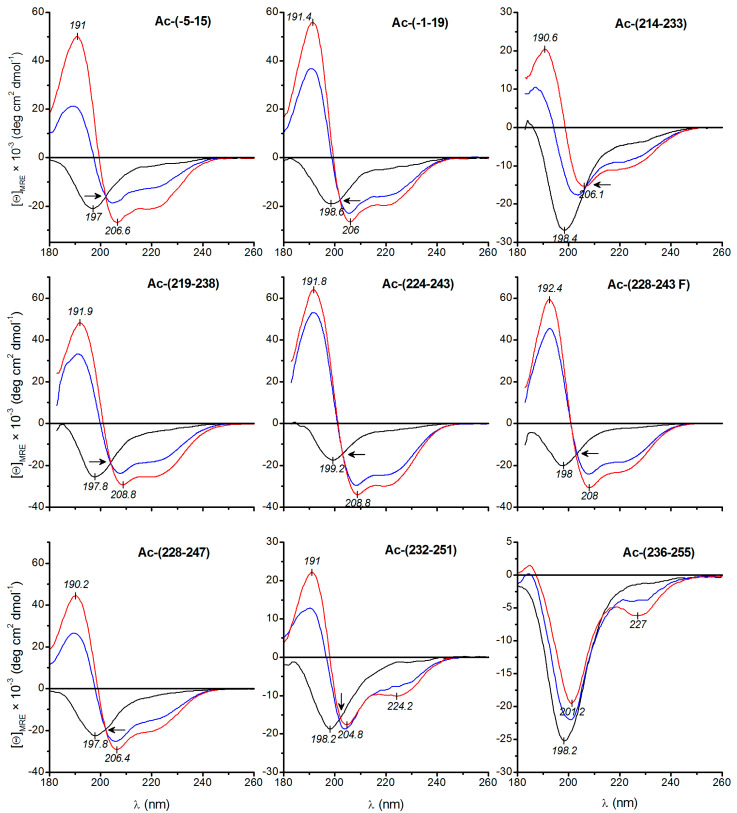
Far-UV CD spectra of HSV peptides measured in water (black), TFE (red) and water:TFE (1:1 v/v) mixture. Arrows denote isodichroic points at λ ~203 nm. For Ac-(214–233), the isodichroic point is shifted to λ = 206 nm.

Electronic circular dichroism (ECD) is a widely employed, non-destructive spectroscopic technique for rapid evaluation of the secondary (far-UV region) and tertiary (near-UV region) structure of peptides and proteins [29]. Therefore, ECD spectroscopy measurements were performed in the far-UV region on selected acetylated HSV-1 gD peptides in TFE, water and TFE-water 1:1 (*v/v*) solutions. In all cases, the ECD curves obtained in water displayed a negative band centred about λ = 195-200 nm and a less intense negative shoulder above λ = 215 nm (Figure 3). Such a spectral pattern is characteristic of the major contribution of disordered conformational state (Table 2) which is in line with the results of crystallographic studies. The *N*-terminal region of the glycoprotein (residues 7–15 and 24–32) was extended and partly disordered when the gD ectodomain was crystallised alone [9,10]. Similarly, no electron density was observed for residues 251 to 285 [7,9]. It is to be noted that the λ_max_ of some peptides was red shifted by cc. 1-2 nm and the short-wavelength tail of the negative band reached the zero baseline instead of showing a local minimum (Figure 3). In accord to these spectral features, the secondary structure analysis indicated a small helical contribution for Ac-(219–238), Ac-(224–243), Ac-(214–233), and Ac-(-1–19) (Table 2).

In relation to water, ECD curves registered in water:TFE and pure TFE solutions exhibited dramatic alterations for most peptides. The spectra consist of an intense, positive-negative band pair centred at λ ~192 / ~208 nm and a weaker, broad negative band with a λ_min_ between λ = 220–230 nm (Figure 3). According to the coupled oscillator theory, the short-wavelength positive and the longer-wavelength negative ECD peak stem from exciton splitting of the π-π^*^ transition of the amide bonds forming a right-handed helix whereas the negative band at λ = 220–222 nm is of n-π^*^ origin [29]. This pattern clearly indicates that the peptides are stabilised in helical conformation which is the consequence of the helix promoting effect of the membrane-mimetic TFE [30,31]. Therefore, it can be proposed that the helical conformations observed here resemble those assumed by the peptide in a cell membrane.

Superimposition of the CD curves obtained in water, TFE and their 1:1 mixture shows a clean conserved isodichroic point near λ = 203 nm for most peptides (Figure 3), which is indicative of a two-state transition from a disordered to highly helical conformation [31].

In stark contrast to the other peptides, the ECD spectrum of Ac-(236–255) reflected only minor conformational changes upon increase of the TFE concentration indicating no ordered secondary structure even in pure TFE solution (Figure 3). Presumably, this result is related to the abundance of the strongly disorder-promoting proline residues in the peptide sequence (Table 2).

Ac-(224–243), Ac-(231–243 F) and Ac-(228–247) showed the highest α-helical content (80–90%) suggesting that these sequences readily adopt helical conformation upon interaction with lipid membranes (Table 2). The helical content of the peptides in different solvents was also estimated employing the simple equation formulated by Chen and Yang: *f*_H_ = -([Θ]_222_ + 2340)/30,300 where *f*_H_ the fraction of helix and [Θ]_222_ is the measured mean residual ellipticity at 222 nm [32]. It seems that the helical fractions obtained correlate favourably with the results of the curve fitting procedure (Table 2).

**Figure 4:**
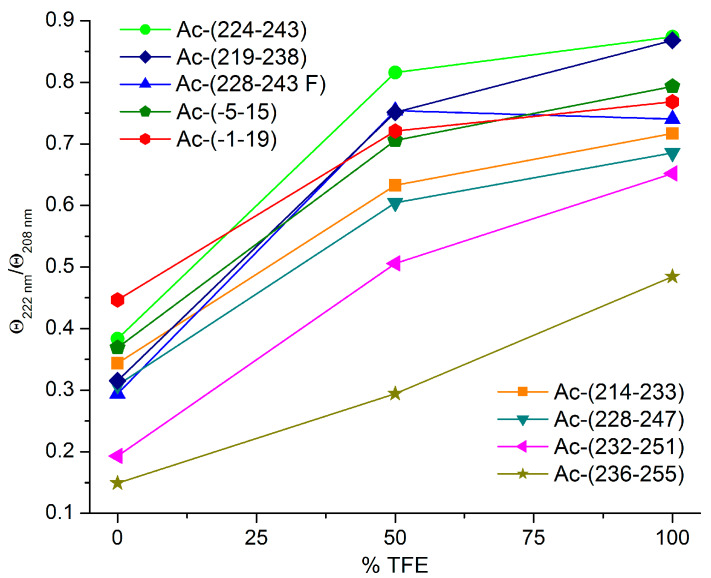
Ratio of Θ_222 nm_/Θ_208 nm_ as a function of TFE concentration for acetylated HSV peptides.

Based on the visual comparison of the ECD curves taken in TFE and TFE-water mixture, the greatest helical propensity can be predicted for Ac-(224–243), Ac-(228–243 F), Ac-(228–247) and Ac-(219–238) (Figure 3). The ECD magnitudes of these peptides are close to each other in these solvents. The helical propensity can be further evaluated by the calculation of the ratio of mean residue ellipticities, Θ_222 nm_/Θ_208 nm_, which is a useful diagnostic parameter. For single stranded α-helices, this value falls between 0.8 and 0.95, whereas above 1 it is symptomatic to the presence of coiled-coil motifs [33]. For HSV peptides showing helical folding, this ratio is ranged within 0.6–0.9 indicating helix formation but not the intermolecular association of the chains (Figure 4).

The peptide structure was also analysed using the PEP-FOLD3 (PEP-FOLD server 2017) service [21,34,35]. PEP-FOLD3 is a de novo approach for predicting the 3D structure, based on structural alphabet (SA) letters to describe the conformations of four consecutive residues followed by the coupling of the predicted series of SA letters to a greedy algorithm and a coarse-grained force field [36,37]. One hundred simulations were run for all sequences. The PEP-FOLD3 results can be interpreted as the propensity of an amino acid in the sequence to be in a helical, random coil or extended strand structure. The structural results of the PEP-FOLD algorithm along with predicted 3D conformations are also shown in Figures S13–S15.

Although PEP-FOLD3 predicts secondary structure propensity based on NMR structures recorded mostly in aqueous solutions [37], the resulting predicted structures for 20mer peptides within region 214–255 correspond with the structure of the regions in the protein, and also with the structure measured by ECD in TFE or aqueous TFE, but not in water (Figures S14 and S15). Region 219–238 is an exception is this region, its predicted structure displays only minor helical content while in the protein larger portion of it adopts helical structure. In the case of the *N*-terminal -5–15 and -1–19 region the high helix-content predicted corresponds to ECD measurements in TFE or aqueous TFE, but not to the known 3D structure (Figure S13). It must be noted that structure 1JMA (HSV-1 gD – HVEM complex) is the only structure available that contains this part of the protein, and in this structure the region 7–17 is in complex with the HVEM. In all other known HSV-1 gD structures this region is invisible due to its unordered conformation.

### 3.3. Cellular Uptake and Intracellular Localisation of HSV-1 gD Cf-Peptides

HSV-1 establishes primary infection in epithelial cells through entry at skin and mucosal sites. In addition to replication at the peripheral epithelium, HSV-1 is able to establish infections in the peripheral nervous system. Neuronal cells harbour the virus in latent phase, and these cells play a pivotal role in persistent infection and cannot be ignored. HSV-1 can reactivate at any time in response to stimuli (physical, emotional stress or compromised immunity) [38,39]. Therefore, neuronal cell line can be employed as a suitable model to investigate the main features of the designed carrier peptides. Internalisation of the peptides was measured on SH-SY5Y human neuroblastoma culture [25,26,27,28], which was chosen as a neuronal host cell model [38,39,40,41].

**Figure 5:**
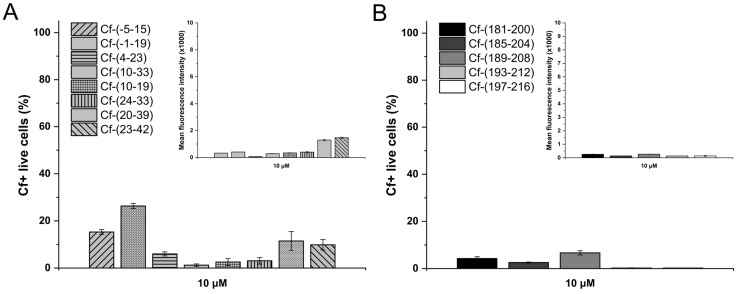
Internalisation of synthetic Cf-HSV-1 gD peptides into SH-SY5Y neuroblastoma cells measured by flow cytometry. (**A**) Internalisation of Set I peptides at c = 10 µM, (**B**) internalisation of Set II peptides at c = 10 µM. Main graphs show the percentage of Cf-positive live cells, inserts show the mean fluorescence intensity. Graphs were plotted using Origin 2018 (OriginLab, Northampton, MA, USA).

**Figure 6:**
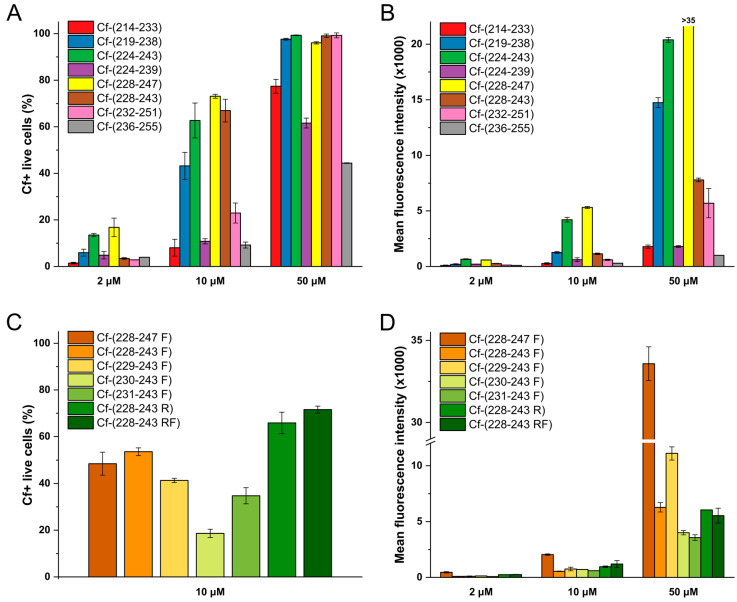
Internalisation of synthetic Cf-HSV-1 gD peptides into SH-SY5Y neuroblastoma cells measured by flow cytometry. (**A**) Internalisation of Set III peptides at c = 2, 10 and 50 µM, percentage of Cf-positive live cells, (**B**) internalisation of Set III peptides at c = 2, 10 and 50 µM, mean fluorescence intensity (**C**) internalisation of Set IV peptides at c = 10 µM, percentage of Cf-positive live cells, (**D**) internalisation of Set IV peptides at c = 10 µM, mean fluorescence intensity. Graphs were plotted using Origin 2018 (OriginLab, Northampton, MA, USA).

Measurements using BD LSR II flow cytometer revealed a concentration-dependent internalisation of the Cf-HSV peptides. The peptide derivatives internalised into host cell model SH-SY5Y human neuroblastoma cells in a dose-dependent manner, characterised by the enhanced intracellular fluorescence measured by BD LSR II flow cytometer. The percentage of Cf-positive (Cf+) live cells with standard error of the mean is demonstrated in the Supplementary Information (Figure S16). Cellular uptake at 10 µM concentration is presented in Figure 5 and Figure 6.

The cell viability was also assessed by flow cytometry using propidium iodide (PI) exclusion method. PI is a membrane impermeant dye that is excluded from viable cells. PI penetrates the damaged, permeable membranes of non-viable cells and it is widely used for determination of membrane integrity and quantification of cell viability. Relative viability after 3 h treatment with Cf-HSV-1 gD Set IV peptides (as representatives) compared to untreated cells is shown in Figure S17. Relative viability of the cells was above 85% for each Cf-peptide.

Significant differences have been observed between the cellular uptake of individual peptides. In the manner of IC_50_ calculations, the UC_50_ value was introduced for a numeric representation of the uptake. UC_50_ is the interpolated concentration required for intracellular fluorescence in 50% of the cells (Table 1). For the interpolation of UC_50_ values see Supplementary Information Figure S18. UC_50_ determination required higher treating concentration (250 μM) in the case of some peptides (mainly Set II peptides, also Cf-(4–23) from Set I and Cf-(236–255) from Set III) in Table 1 to identify these peptides, see UC_50_ values higher than 50 μM.

In Set I, three overlapping 20mer peptides were chosen from the N-terminal of the HSV-1 gD glycoprotein which are only partly visible on the complex structures of either HSV-1 gD – nectin or HSV-1 gD – HVEM. Further two N-terminal peptide sequences based on the structure of the HSV-1 gD – nectin complex (20–39, 23–42), and a sequence based on the structure of the HSV-1 gD – HVEM complex (10–33) and its N- and C-terminal decamers were prepared and studied for their cellular uptake (Figure 5A, Figure S16a,b). Except for peptide Cf-(4–23) (UC_50_ ~ 90 μM), these entered SY5Y cells with UC_50_ values between 17 and 40 μM. Peptide Cf-(4–23) showed low internalisation even at c = 250 μM, 63% Cf positive live cells. The highest internalisation ability among these peptides was observed in case of Cf-(-1–19) peptide (UC_50_ = 17.5 μM), at c = 10 μM the Cf-positive live cells exceeded 25%. Peptide Cf-(10–33) and its N- and C-terminal decamers showed mediocre internalisation (UC_50_ values were between 30 and 40 μM). This peptide covers two regions in close proximity with HVEM connected by a hairpin structure in the HSV-1 gD – HVEM complex. Both the N-terminal Cf-(10–19) and C-terminal Cf-(24–33) peptides internalised with slightly higher efficiency than the parent peptide, Cf-(10–33). Higher fluorescence intensity was observed, but in smaller percentage of cells in case of the possibly nectin-1 binding Cf-(20–39) and Cf-(23–42) peptides.

A series of 20mer peptides (Set II) from the central part of HSV-1 gD (181–216) was found to have very poor internalisation efficiency, with the exception of Cf-(181–200) with mediocre internalisation (UC_50_ = 36.8 μM), their UC_50_ values were above 50 μM, in case of Cf-(193–212) and Cf-(197–216) above 100 μM (Figure 5B, Figure S16c,d). Even in c = 250 μM, the percentage of Cf positive live cells was generally under 80%.

When the nectin-1 binding *C*-terminal region of HSV-1 gD (Set III) was analysed with overlapping 20mer peptides, we found peptides with strong internalisation ability (Figure 6A,B). The highest cellular uptake among them was observed in case of Cf-(228–247), followed by Cf-(224–243) and Cf-(219–238) (UC_50_ = 4.7, 6.6 and 12.2 μM, respectively). Even in c = 2 μM concentration these peptides resulted in 15–20% of Cf-positive live cells. Towards both *N*- and *C*-termini the internalisation gradually decreased. From these results the sequence 228–243, the common sequence between 228–247 and 224–243 has been deduced as participating most in the cellular entry, and peptide Cf-(228–243) proved to be also effective with UC_50_ = 6.5 μM, although the mean fluorescence intensity was lower. It should be noted that the fluorescence intensity of this peptide, measured by fluorimetry, was also lower. The common sequence between the second and third most effectively internalising peptides, 224–239 was also examined, but Cf-(224–239) was found to have lower internalising ability (UC_50_ = 23.7 μM) than either of its parent peptides. Therefore, peptides derived from the 228–243 sequence were examined further.

Examination of the cellular entry ability of set IV peptides – modified and/or truncated variants of Cf-(228–247) – revealed that Trp^241^ may be substituted for Phe without significant loss of internalisation ability (Cf-(228–247): 4.7 μM vs. Cf-(228–247 F): 10.5 μM, Cf-(224–243): 6.5 μM vs. Cf-(228–243 F): 8.9 μM UC_50_). *N*-terminally truncated versions of peptide Cf-(228–243 F) have also been examined, but we have observed that their cellular uptake was lower than that of the parent peptide, resulting in UC_50_ values between 12 and 20 μM (Figure 6C,D, Figure S16e,f).

Not only Trp^241^Phe, but also Lys^237^Arg exchange was performed systematically in the 228–243 sequence. As seen in Figure 6C,D and Figure S16e,f, these modifications did not decrease the internalisation rate of these peptides (UC_50_ values were between 6 and 9 μM).

The cellular uptake of HSV-1 gD peptides has been compared with a tuftsin peptide (OT10) and a cell penetrating peptide, Penetratin. OT10 is a non-membrane-active, receptor binding peptide, which represents cationic character. Penetratin is a membrane-active, amphiphilic cell penetrating peptide (cpp) with superior penetration ability and low in vitro toxicity on human cells.

Tuftsin is a natural peptide produced by enzymatic cleavage of the Fc-domain of the heavy chain of immunoglobulin G. During the past decade, a new group of sequential oligopeptide carriers with discrete molecular masses has been developed in our laboratory: these oligotuftsin derivatives consisting of tandem pentapeptide repeat unit [TKPKG]_n_ (n = 2, 4, 6 and 8) are based on the canine tuftsin sequence TKPK. These compounds are nontoxic and non-immunogenic and are developed to be effective tuftsin-receptor specific carrier peptides [42,43] to deliver, e.g., antitubercular drug candidates into monocytic host cells [20,44]. In this study, we have compared the internalising ability of Cf-TKPKGTKPKG (Cf-OT10) peptide with that of HSV-1 gD peptides on SH-SY5Y cells (comparison of Set II peptides and OT10 is shown in Figure S19). There are no tuftsin receptors on SH-SY5Y cells [45]. Although in large concentration (c = 50 µM) OT10 shows effective cellular uptake, though not reaching 100%, in low c = 10 µM concentration it did not reach the level of internalisation of HSV peptides even with moderate uptake level.

Cpps and their numerous synthetic derivatives are the focus of research and potential drug delivery applications. These cpps are promising chemical helpers to transport non-permeable drugs into live cells [17,46,47,48,49,50]. One of the most studied cpp is Penetratin, which was derived from the sequence of HIV transactivator protein [51] and the third helix of the homeodomain of *Drosophila* Antennapedia protein [52] Peptide Cf-(228–247) internalised in a comparable way with Cf-Penetratin (Figure S20).

**Figure 7:**
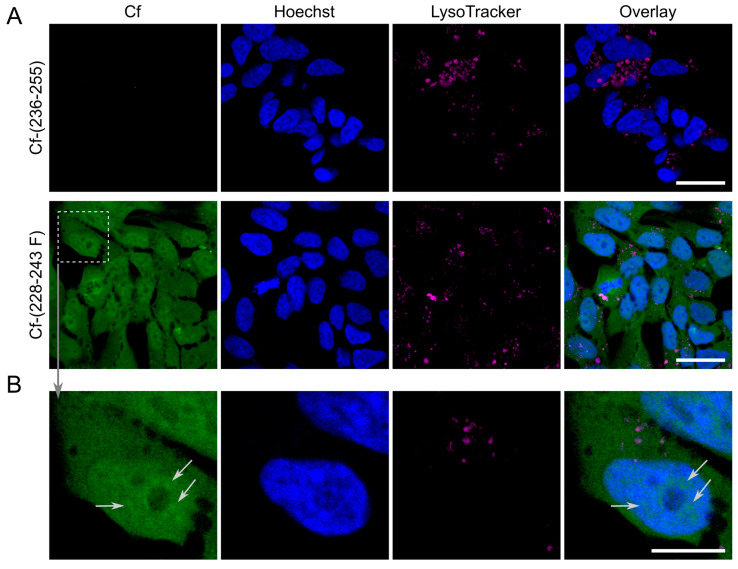
(**A**) Intracellular localisation of peptides Cf-(228–243 F) and Cf-(236–255) by confocal laser scanning microscopy. Cells were incubated for 3 h with Cf-labelled peptides (c = 25 µM, green), lysosomes were labelled with LysoTracker Deep Red (magenta), nuclei were stained by Hoechst 33342 (blue). (**B**) Enlargements present ubiquitous distribution of peptide Cf-(228–243 F). Scale bar represents 20 µm (**A**) and 10 µm (**B**).

Parallel with flow cytometry measurements to assess qualitative information regarding the subcellular localisation, the internalised Cf-(228–243 F) (with UC_50_ = 8.9 µM) was imaged by confocal laser scanning microscopy. To visualise intracellular localisation of the Cf-peptides, LysoTracker^TM^ Deep Red was used for lysosome and Hoechst 33342 for nuclear staining. As a peptide with low internalisation rate, Cf-(236–255) (UC_50_ = 65.7 µM) was also investigated. The experiment was carried out with 3 h incubation time and only representative images are shown (Figure 7). As was expected based on the results of flow cytometry measurements, in the case of peptide Cf-(236–255) no internalisation occurred, no trace of fluorescent signal was observed in the cytosol and in lysosomal compartments (Figure 7A, upper panels). Peptide Cf-(228–243 F) could be imaged in the cytosol and the nucleus, but there is no co-localisation with lysosomal staining (Figure 7A, lower panels). This suggests that there is no vesicular transport involved in the uptake of the Cf-peptide (no direct co-localisation with lysosomes). The Cf-peptide internalises and displays a ubiquitous distribution in the cytosol and in the nucleus as well. Since the Cf signal only partially co-localises with the lysosomes this pathway could be deemed negligible (presented at Figure 7B, enlargements).

## 4. Discussion

**Figure 8:**
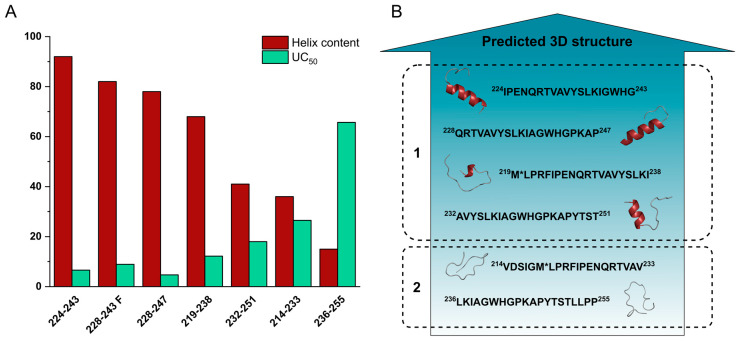
(**A**) Comparison of the helix content in TFE solution of acetylated HSV peptides based on ECD; calculated with the formula of Chen and Yang [32] (Table 2) with their respective UC_50_ values of Cf-peptides, data were obtained from cellular uptake studies (Table 1). (**B**) PEP-FOLD3 predicted secondary structure of HSV peptides (Figures S14 and S15), the prediction method refers structures in mostly aqueous solutions [21,37].

The ECD spectroscopic results are in good agreement with the cell internalisation data presented. The best UC_50_ values (UC_50_ < 10 μM) were obtained for peptides Cf-(224–243) and Cf-(228–247) whose acetylated counterparts exhibit pronounced helical folding in the membrane mimicking solvent TFE, also peptide Cf-(228–243) and its W→F and K→R derivatives, of which Ac-(228–243 F) showed similar structural characteristics to the above two peptides. Conversely, sequences associated with little helical character even in pure TFE such as Ac-(214–233), Ac-(232–251) and Ac-(236–255) (Figure 8, Table 2) showed decreasing internalisation rates. Cf-peptide of the 219–238 sequence has relatively high, but not outstanding internalization rate, while Ac-(219–238) also showed lower helical content than peptides of the 224–247 region. The relationship between the helical propensity of the peptides and their cellular uptake is demonstrated in Figure 8A. Among the peptides in the nectin-1-binding region of HSV-1 gD, we found that the higher the helix content in TFE is, the higher the cellular entry rate is (the lower the UC_50_ value). For visualisation, in Figure 8B the UC_50_ values of Set III peptides are contrasted with their PEP-FOLD3 secondary structure prediction structures.

A possible explanation of the differences between the peptides’ uptake rate may be based on mainly conformational features. Our data suggest that the helicity of the peptides is the most important factor in the process of internalisation. This hypothesis can be supported by the above presented parallel between internalisation (quantified by UC_50_) and helical content (Figure 8).

**Figure 9:**
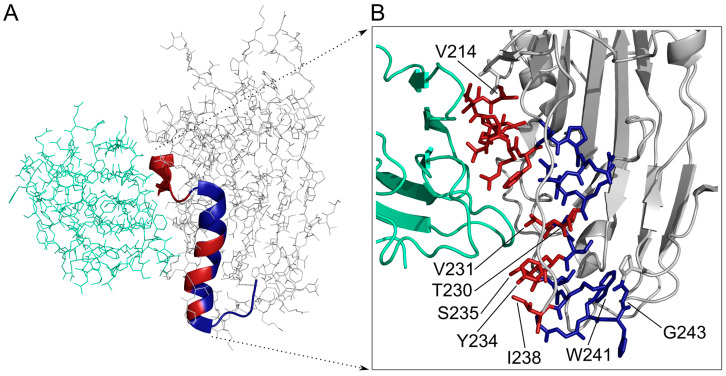
3D structure of HSV-1 gD glycoprotein (grey) in complex with nectin-1 (ocean green). (**A**) the ribbon indicates the region covered by Set III peptides, red parts participate in the HSV-1 gD – nectin-1 interaction. (**B**) Details of the binding surface with HSV-1 gD 214–243 region are in stick representation, red residues are in contact with nectin-1. After the strongly binding 214–223 segment (red) the non-contacting ^224^IPENQR^229^ segment can be seen (blue), then 230TVAVYSLKI^238^ helical region with alternating contacting and non-contacting residues. Segment ^240^GWHG^243^ (blue) is folding back on the helical region.

The most effectively internalising peptides contain only 5 residues of HSV-1 gD (^230^TV^231^, ^234^YS^235^ and I^238^) which are in close contact with nectin-1 according to the crystal structure of the complex (Figure 9A, red parts of the helix) and not the 214–227 region, which has several more contacting residues in a loop and a β-strand region [4,5]. On the other hand, interaction of parts of this region with nectin-1 may also promote the internalisation of certain peptides. Therefore, we have analysed the internalisation of peptides from the 214–243 region in the light of the localisation of this region in the 3D structure of HSV-1 gD and nectin-1 complex (Figure 9B).

Peptides Cf-(228–43), Cf-(224–243) and the 16mer Cf-(228–243) deduced from their sequence all showed higher internalising rate than Cf-(219–238) (UC_50_ = 12.2 μM), therefore the sequence ^239^AGWHG^243^ probably plays a role in the sequence requirements for effective cellular uptake. According to the HSV-1 gD – nectin-1 complex X-ray structure [6,9], ^239^AGWHG^243^ is not participating in this interaction, but ^240^GWHG^243^ folds back on the 234–239 helical region (Figure 9B) and may promote or stabilise the suitable peptide conformation for internalisation. Another 16mer peptide, Cf-(224–239), containing the common sequence of Cf-(224–243) and Cf-(219–238) was expected to have similar uptake ability to its parent peptides, but the determined internalisation level was significantly lower (UC_50_ = 23.7 μM). Thus, the presence of ^219^MLPRF^223^ markedly increased the cellular uptake of peptide Cf-(219–238) compared to the 16mer. These residues in the complex structure form an extended conformation in close contact with the C”C’CFG β-sheet of nectin-1 (Figure 9B). It can be hypothesised that in case of peptides containing this region the cellular entry is mediated by a possible attachment to nectin-1, resulting in accumulation of the peptide on the cell surface, prior to the internalisation. The nucleus is one of the most important organelles in eukaryotic cells. Targeted nuclear delivery is an increasing and important area of drug delivery and gene therapy related research. Our data suggest that peptide Cf-(228–243 F) possesses nuclear internalisation ability. This phenomenon can be explained by the finding that the region 228–247 contains nuclear localisation signal pattern (WHGPKA) and shares a homologue sequence with the ^52^WTGVEA^57^ sequence of the HIV-1 Vpr52–93 sequence which was proven to internalise [53]. Using the approach described by Kanduc [54], we also determined a short sequence within this peptide (KIA) which is homologous with adenoviral Ad3 fibre protein derived nucleus targeting peptide.

The topic of our next study will be to assess the exact mechanism of the cellular entry process and the intracellular trafficking of the peptides.

## 5. Conclusions

Ligand-targeted delivery can improve the cellular uptake of different cargoes (i.e., drugs) through specific binding to receptors that are expressed or even overexpressed on a particular target cell surface. Large number of targeted ligands have been discovered for cell directed delivery, such as peptides and antibodies. Therefore, identifying potential new carrier peptides from viral proteins is a novel available tool. These peptides possess favourable internalisation properties and they can be promising candidates in the development of innovative delivery vehicles and reliable alternatives to other type of carrier peptides. In this study, we have demonstrated that 16mer and 20mer peptides derived from the nectin-1 binding region of the HSV-1 gD glycoprotein are able to internalise in SH-SY5Y neuroblastoma cells with high efficiency. The most potent peptides are corresponding to the ^224^IPENQRTVAVYSLKIAGWHGPKAP^247^ sequence of HSV-1 gD, and most of that part has helical conformation within the protein. These peptides show a tendency of forming helical structure in lipomimetic TFE solution as well. Overall, it can be concluded that the disorder-to-helix conformational transition of HSV-1 gD peptides is a decisive structural feature for their cell entry mediated by biomembrane or nectin-1 interactions. As lead sequences for further studies ^228^QRTVAVYSLKIAGWHGPKAP^247^ or ^228^QRTVAVYSLKIAGWHG^243^ were indicated, with Lys^237^Arg and/or Trp^241^Phe substitutions for stability and side-reaction free conjugation of cargo molecules.

We plan a comprehensive study for evaluating the cellular entry mechanism and intracellular trafficking of HSV-1 gD peptides.

## Figures and Tables

**Table 1:** Analytical evaluation of Cf-labelled HSV-1 gD synthetic peptides.

Code	Set	Sequence	Mw calc / meas^a^	UC_50_^b^
Cf-(-5–15)	I	Cf-^-5^HGVRGKYALADASLKMADPN^15^	2471.7/2471.7	21.1
Cf-(-1–19)		Cf-^-1^GKYALADASLKMADPNRFRG^19^	2538.8/2539.0	17.5
Cf-(4–23)		Cf-^4^LADASLKMADPNRFRGKDLP^23^	2572.8/2572.8	91.9
Cf-(10–33)		Cf-^10^KMADPNRFRGKDLPVLDQLTDPPG^33^	3038.4/3038.5	40.3
Cf-(10–19)		Cf-^10^KMADPNRFRG^19^	1548.7/1548.6	35.1
Cf-(24–33)		Cf-^24^VLDQLTDPPG^33^	1411.5/1410.8	31.6
Cf-(20–39)		Cf-^20^KDLPVLDQLTDPPGVRRVYH^39^	2676.0/2675.1	23.0
Cf-(23–42)		Cf-^23^PVLDQLTDPPGVRRVYHIQA^42^	2631.9/2631.3	23.1
Cf-(181–200)	II	Cf-^181^LEHRAKGSCKYALPLRIPPS^220^	2665.1/2665.2	36.8
Cf-(185–204)		Cf-^185^AKGSCKYALPLRIPPSACLS^204^	2575.0/2575.5	92.9
Cf-(189–208)		Cf-^189^CKYALPLRIPPSACLSPQAY^208^	2691.1/2691.0	96.6
Cf-(193–212)		Cf-^193^LPLRIPPSACLSPQAYQQGV^212^	2566.9/2566.9	115.4
Cf-(197–216)		Cf-^197^IPPSACLSPQAYQQGVTVDS^216^	2489.7/2489.5	108.3
Cf-(214–233) ^c^	III	Cf-^214^VDSIGM*LPRFIPENQRTVAV^233^	2581.9/2581.2	26.5
Cf-(219–238)		Cf-^219^M*LPRFIPENQRTVAVYSLKI^238^	2715.1/2713.8	12.2
Cf-(224–243)		Cf-^224^IPENQRTVAVYSLKIAGWHG^243^	2596.8/2596.1	6.6
Cf-(224–239)		Cf-^224^IPENQRTVAVYSLKIA^239^	2159.4/2159.0	23.7
Cf-(228–247)		Cf-^228^QRTVAVYSLKIAGWHGPKAP^247^	2536.9/2537.2	4.7
Cf-(228–243)		Cf-^228^QRTVAVYSLKIAGWHG^243^	2143.4/2143.3	6.5
Cf-(232–251)		Cf-^232^AVYSLKIAGWHGPKAPYTST^251^	2504.8/2505.0	18.0
Cf-(236–255)		Cf-^236^LKIAGWHGPKAPYTSTLLPP^255^	2504.9/2505.0	65.7
Cf-(228–247 F) ^c^	IV	Cf-^228^QRTVAVYSLKIAGFHGPKAP^247^	2497.8/2498.0	10.5
Cf-(228–243 F)		Cf-^228^QRTVAVYSLKIAGFHG^243^	2104.3/2104.4	8.9
Cf-(229–243 F)		Cf-^229^RTVAVYSLKIAGFHG^243^	1976.2/1975.6	12.7
Cf-(230–243 F)		Cf-^230^TVAVYSLKIAGFHG^243^	1820.0/1819.1	19.1
Cf-(231–243 F)		Cf-^231^VAVYSLKIAGFHG^243^	1718.9/1718.5	14.7
Cf-(228–243 R)		Cf-^228^QRTVAVYSLRIAGWHG^243^	2171.4/2171.3	6.7
Cf-(228–243 RF)		Cf-^228^QRTVAVYSLRIAGFHG^243^	2132.3/2132.0	6.0

All Cf-peptides were amidated on the *C*-terminus and isolated as TFA salt. ^a^ Bruker Esquire 3000+ ESI MS mass spectrometer. ^b^ UC_50_: interpolated concentration required for intracellular fluorescence in 50% of the cell. ^c^ M*: methionine norleucine exchange, F: Trp^241^Phe, R: Lys^237^Arg exchange.

**Table 2:** Estimation of the secondary structure fraction of acetylated HSV peptides. Far-UV CD spectroscopic data were analysed by using the MS Excel version of the PEPFIT program. Values in bracket show the helical content obtained with the formula of Chen and Yang [32]. M*: methionine residue has been substituted by norleucine.

	H_2_O	H_2_O:TFE	TFE	H_2_O	H_2_O:TFE	TFE
**^-5^** **HGVRGKYALADASLKMADPN^15^**				**^-1^** **GKYALADASLKMADPNRF^19^**		
α-helix	0 (3)	39 (33)	66 (60)	8 (7)	50 (42)	70 (56)
β-sheet	15	0	0	4	0	0
disordered	70	30	2	64	10	0
turn	15	31	32	24	40	30
*R* ^2^	0.9833	0.9961	0.9952	0.9931	0.9909	0.9804
**^214^** VDSIGM*LPRFIPENQRTVAV^233^				**^219^** M^*^LPRFIPENQRTVAVYSLKI^238^		
α-helix	6 (7)	18 (22)	36 (28)	8 (5)	48 (51)	68 (76)
β-sheet	0	8	0	0	20	19
disordered	74	40	36	78	26	2
turn	20	34	28	14	6	11
*R* ^2^	0.9819	0.9957	0.9916	0.9810	0.9954	0.9921
**^224^** **IPENQRTVAVYSLKIAGWHG^243^**				**^228^** **QRTVAVYSLKIAGWHGPKAP^247^**		
α-helix	8 (3)	73 (72)	92 (89)	0 (3)	48 (41)	78 (57)
β-sheet	22	20	8	0	0	0
disordered	70	7	0	73	38	0
turn	0	0	0	27	14	22
*R* ^2^	0.9747	0.9950	0.9957	0.9863	0.9945	0.9939
**^232^** **AVYSLKIAGWHGPKAPYTST^251^**				**^236^** **LKIAGWHGPKAPYTSTLLPP^255^**		
α-helix	0 (0)	32 (18)	41 (25)	0 (0)	6 (5)	15 (10)
β-sheet	0	8	0	0	4	0
disordered	48	52	42	60	60	64
turn	52	8	17	40	30	21
*R* ^2^	0.9950	0.9800	0.9776	0.9767	0.9920	0.9942
**^228^** **QRTVAVYSLKIAGFHG^243^**						
α-helix	0 (0)	56 (52)	82 (67)			
β-sheet	20	20	2			
disordered	68	10	4			
turn	12	14	12			
*R* ^2^	0.9841	0.9889	0.9872			

## Supplementary Materials

The following are available online at https://www.mdpi.com/2218-273X/10/5/721/s1, Figure S1. ESI-MS spectra of Set I peptides; Figure S2. ESI-MS spectra of Set II peptides; Figure S3. ESI-MS spectra of Set III peptides; Figure S4. ESI-MS spectra of Set IV peptides; Table S1. Analytical data of acetylated HSV-1 gD peptides; Figure S5. RP-HPLC chromatograms and ESI-MS spectra of Cf-HSV peptides prepared manually on Mimotopes lanterns; Figure S6. RP-HPLC chromatograms and ESI-MS spectra of Cf-HSV peptides prepared by Syro-I automated peptide synthesiser; Figure S7. RP-HPLC chromatograms and ESI-MS spectra of Cf-HSV peptides prepared manually on Rink-amide MBHA resin; Figure S8. Fluorescence spectra of Cf-HSV-1 gD peptides at pH = 4.0; Figure S9. Fluorescence spectra of Cf-HSV-1 gD peptides at pH = 5.0; Figure S10. Fluorescence spectra of Cf-HSV-1 gD peptides at pH = 6.0; Figure S11. Fluorescence spectra of Cf-HSV-1 gD peptides at pH = 7.0; Figure S12. Stability study of Cf-(236–255) and Cf-(228–247), followed by RP-HPLC; Figure S13. PEP-FOLD3 prediction of region HSV-(-5–19); Figure S14. PEP-FOLD3 prediction of region HSV-(214–243); Figure S15. PEP-FOLD3 prediction of region HSV-(228–255); Figure S16. Internalisation of synthetic Cf-HSV-1 gD peptides into SH-SY5Y neuroblastoma cells measured by flow cytometry; Figure S17. Relative viability (Set IV); Figure S18. Interpolation using MicroSoft ExCel to determine UC_50_ values; Figure S19. Comparison of the internalisation of Cf-HSV and Cf-oligotuftsin peptides into SH-SY5Y neuroblastoma cells measured by flow cytometry. Figure S20. Comparison of the internalisation of Cf-(228–247 and Cf-(228–243 F) peptides with Cf-Penetratin into SH-SY5Y neuroblastoma cells measured by flow cytometry.
