# Supplementary material for: Tailoring Uptake Efficacy of HSV-1 gD Tailoring Uptake Efficacy of Hsv-1 GD Derived Carrier Peptides
Source: Biomolecules. 2020 May 6;10(5):721. doi: 10.3390/biom10050721 (PMC7277387; doi:10.3390/biom10050721)
Supplement: Supplementary file 1 [file biomolecules-10-00721-s001.pdf]

biomolecules

Article

Tailoring Uptake Efficacy of HSV-1 gD Tailoring Uptake Efficacy of Hsv-1 GD Derived Carrier Peptides

Szilvia Bősze [1], Ferenc Zsila [2], Beáta Biri-Kovács [1,3], Bálint Szeder [4], Zsuzsa Majer [3], Ferenc Hudecz [1,3] and Katalin Uray [1,*]

1 MTA-ELTE Research Group of Peptide Chemistry, 112, P.O. Box 32, H-1518 Budapest, Hungary; bosze@elte.hu (S.B.); beabiri@caesar.elte.hu (B.B.-K.); fhudecz@elte.hu (F.H.); uray@elte.hu (K.U.)
2 Institute of Materials and Environmental Chemistry, Research Centre for Natural Sciences, P.O. Box 286, H-1519 Budapest, Hungary ; zsila.ferenc@ttk.mta.hu
3 Eötvös Loránd University, Institute of Chemistry, Budapest, 112, P.O. Box 32, H-1518 Hungary; majer@chem.elte.hu
4 Institute of Enzymology, Research Centre for Natural Sciences, P.O. Box 286, H-1519 Budapest, Hungary; szederbalint@gmail.com
* Correspondence: E-mail: uray@elte.hu; Tel.: +36-1-3722500/1415

Supplementary information.

Contents

Figure S1. ESI-MS spectra of Set I peptides
Figure S2. ESI-MS spectra of Set II peptides
Figure S3. ESI-MS spectra of Set III peptides
Figure S4. ESI-MS spectra of Set IV peptides
Table S1. Analytical data of acetylated HSV-1 gD peptides
Figure S5. RP-HPLC chromatograms and ESI-MS spectra of Cf-HSV peptides prepared manually on Mimotopes lanterns
Figure S6. RP-HPLC chromatograms and ESI-MS spectra of Cf-HSV peptides prepared by Syro-I automated peptide synthesiser
Figure S7. RP-HPLC chromatograms and ESI-MS spectra of Cf-HSV peptides prepared manually on Rink-amide MBHA resin
Figure S8. Fluorescence spectra of Cf-HSV-1 gD peptides at pH = 4.0
Figure S9. Fluorescence spectra of Cf-HSV-1 gD peptides at pH = 5.0
Figure S10. Fluorescence spectra of Cf-HSV-1 gD peptides at pH = 6.0
Figure S11. Fluorescence spectra of Cf-HSV-1 gD peptides at pH = 7.0
Figure S12. Stability study of Cf-(236–255) and Cf-(228–247), followed by RP-HPLC
Figure S13. PEP-FOLD3 prediction of region HSV-(-5–19)
Figure S14. PEP-FOLD3 prediction of region HSV-(214–243)
Figure S15. PEP-FOLD3 prediction of region HSV-(228–255)
Figure S16. Internalisation of synthetic Cf-HSV-1 gD peptides into SH-SY5Y neuroblastoma cells measured by flow cytometry
Figure S17. Relative viability (Set IV)
Figure S18. Interpolation using MicroSoft ExCel to determine UC_{50} values
Figure S19. Comparison of the internalisation of Cf-HSV and Cf-oligotuftsin peptides into SH-SY5Y neuroblastoma cells measured by flow cytometry.
Figure S20. Comparison of the internalisation of Cf-(228-247 and Cf-(228-243 F) peptides with Cf-Penetratin into SH-SY5Y neuroblastoma cells measured by flow cytometry.

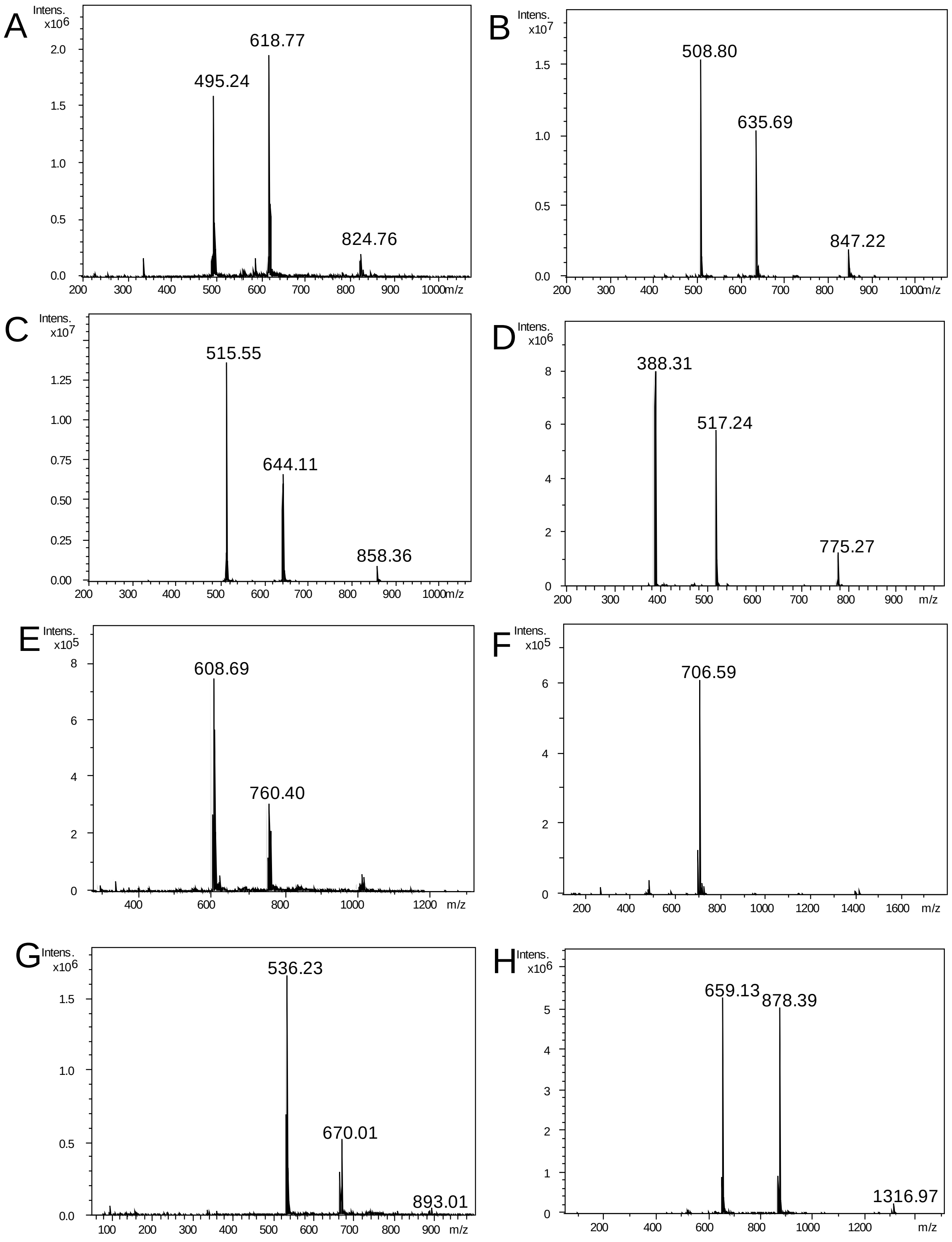

Figure S1. ESI-MS spectra of Set I peptides. (**a**) Cf-(-5–15), (**b**) Cf-(-1–19), (**c**) Cf-(4–23), (**d**) Cf-(10–33), (**e**) Cf-(10–19), (**f**) Cf-(24–33), (**g**) Cf-(20–39), (**h**) Cf-(23–42). ESI-MS was performed on Bruker Esquire 3000 Plus Ion Trap Mass Spectrometer (Bremen, Germany) that operated in continuous sample injection at a 10 μL/min flow rate. Samples were dissolved in 50% acetonitrile–50% water containing 0.1% acetic acid. Mass spectra were recorded in positive mode in the *m*/*z* 50–2000 range.

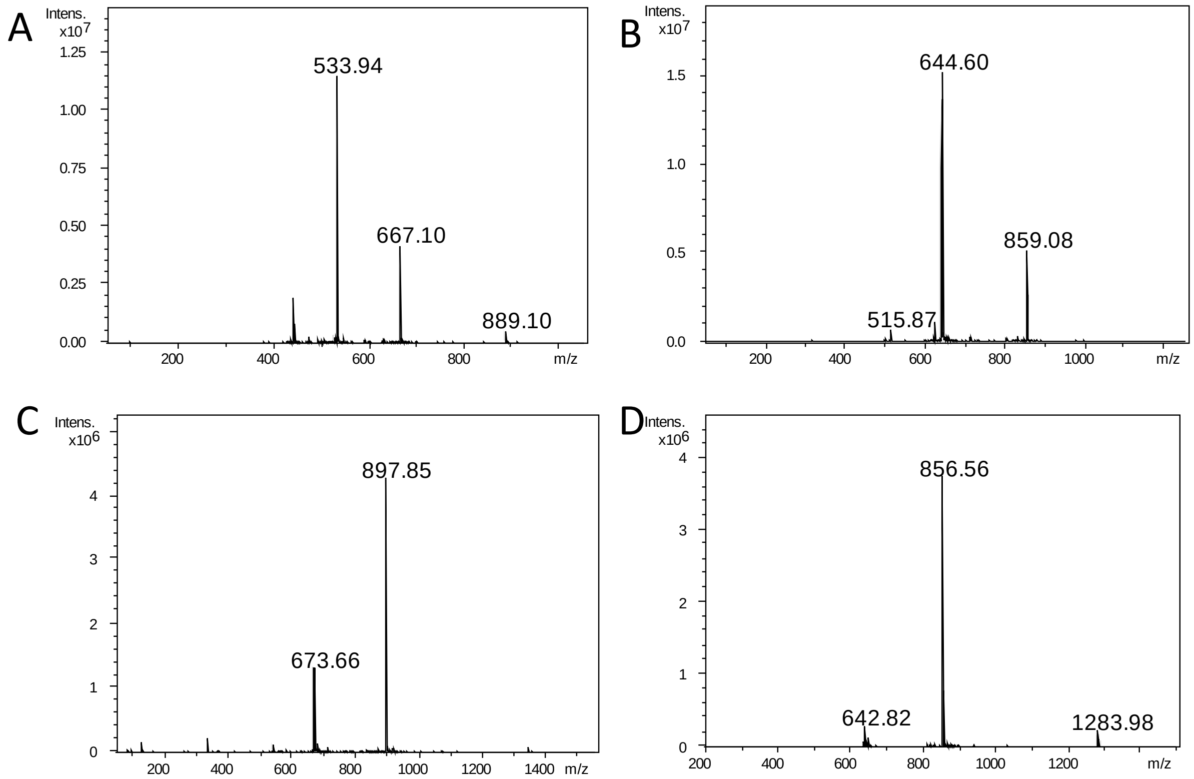

Figure S2. ESI-MS spectra of Set II peptides. (**a**) Cf-(181–200), (**b**) Cf-(185–204), (**c**) Cf-(189–208), (**d**) Cf-(193–212). ESI-MS was performed on Bruker Esquire 3000 Plus Ion Trap Mass Spectrometer (Bremen, Germany) that operated in continuous sample injection at a 10 µL/min flow rate. Samples were dissolved in 50% acetonitrile–50% water containing 0.1% acetic acid. Mass spectra were recorded in positive mode in the *m*/*z* 50–2000 range.

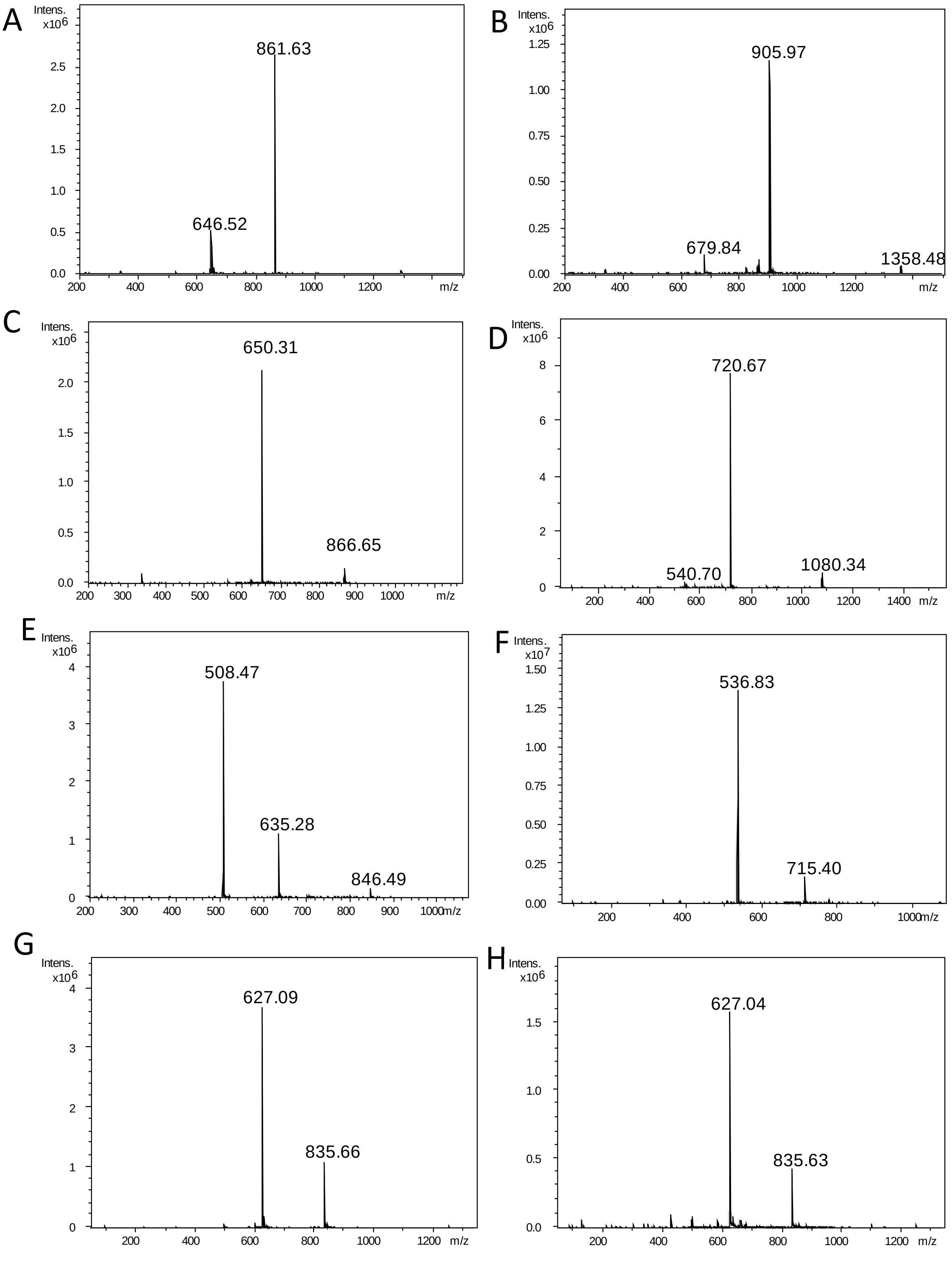

Figure S3. ESI-MS spectra of Set III peptides. (**a**) Cf-(214–233), (**b**) Cf-(219–238), (**c**) Cf-(224–243), (**d**) Cf-(224–239), (**e**) Cf-(228–247), (**f**) Cf-(228–243), (**g**) Cf-(232–251), (**h**) Cf-(236–255). ESI-MS was performed on Bruker Esquire 3000 Plus Ion Trap Mass Spectrometer (Bremen, Germany) that operated in continuous sample injection at a 10 μL/min flow rate. Samples were dissolved in 50% acetonitrile–50% water containing 0.1% acetic acid. Mass spectra were recorded in positive mode in the *m*/*z* 50–2000 range.

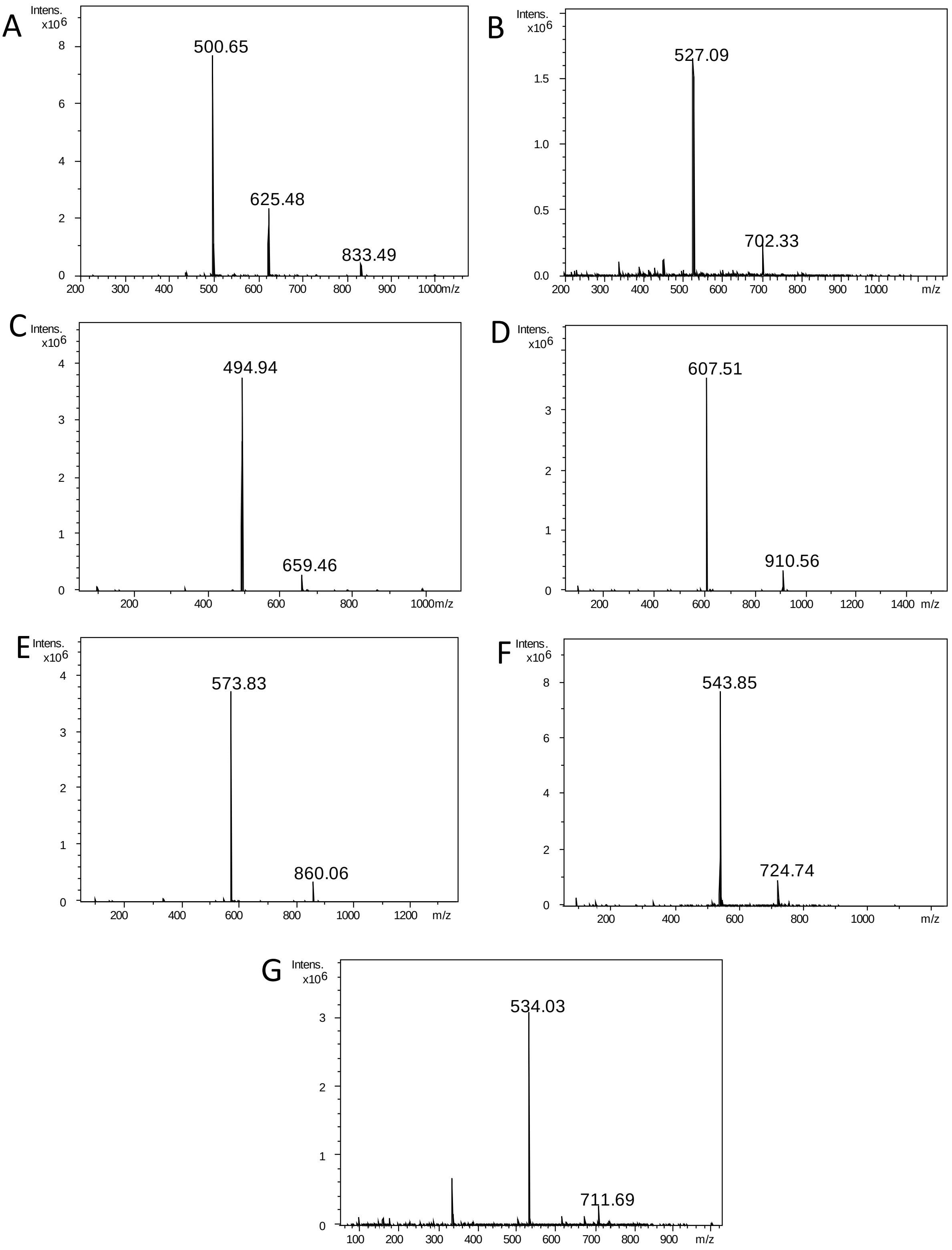

Figure S4. ESI-MS spectra of Set IV peptides. (**a**) Cf-(228–247 F), (**b**) Cf-(228–243 F), (**c**) Cf-(229–243 F), (**d**) Cf-(230–243 F), (**e**) Cf-(231–243 F), (**f**) Cf-(228–243 R), (**g**) Cf-(228–243 RF). ESI-MS was performed on Bruker Esquire 3000 Plus Ion Trap Mass Spectrometer (Bremen, Germany) that operated in continuous sample injection at a 10 μL/min flow rate. Samples were dissolved in 50% acetonitrile–50% water containing 0.1% acetic acid. Mass spectra were recorded in positive mode in the *m*/*z* 50–2000 range.

Table S1. Analytical data of acetylated HSV1 gD peptides.

Code	Sequence	MS calc / meas [a]
Ac-(-5–15)	Ac-$^{-5}$HGVRGKYALADASLKMADPN^{15}	2155.4 / 2155.6
Ac-(-1–19)	Ac-$^{-1}$GKYALADASLKMADPNRFRG^{19}	2222.5 / 2222.7
Ac-(214–233)	Ac-^{214}VDSIGM*LPRFIPENQRTVAV^{233}	2265.6 / 2265.3
Ac-(219–238) [b]	Ac-^{219}M*LPRFIPENQRTVAVYSLKI^{238}	2398.9 / 2400.9
Ac-(224–243)	Ac-^{224}IPENQRTVAVYSLKIAGWHG^{243}	2280.6 / 2280.4
Ac-(228–247)	Ac-^{228}QRTVAVYSLKIAGWHGPKAP^{247}	2220.6 / 2220.9
Ac-(228–243 F) [b]	Ac-^{228}QRTVAVYSLKIAGFHG^{243}	1788.1 / 1787.4
Ac-(232–251)	Ac-^{232}AVYSLKIAGWHGPKAPYTST^{251}	2188.5 / 2188.6
Ac-(236–255)	Ac-^{236}LKIAGWHGPKAPYTSTLLPP^{255}	2188.6 / 2188.6

All peptides were amidated on the *C*-terminus and isolated as TFA salts.

[a] Bruker Esquire 3000+ ESI MS mass spectrometer.

[b] M*: methionine norleucine exchange, F: Trp^{241}Phe exchange.

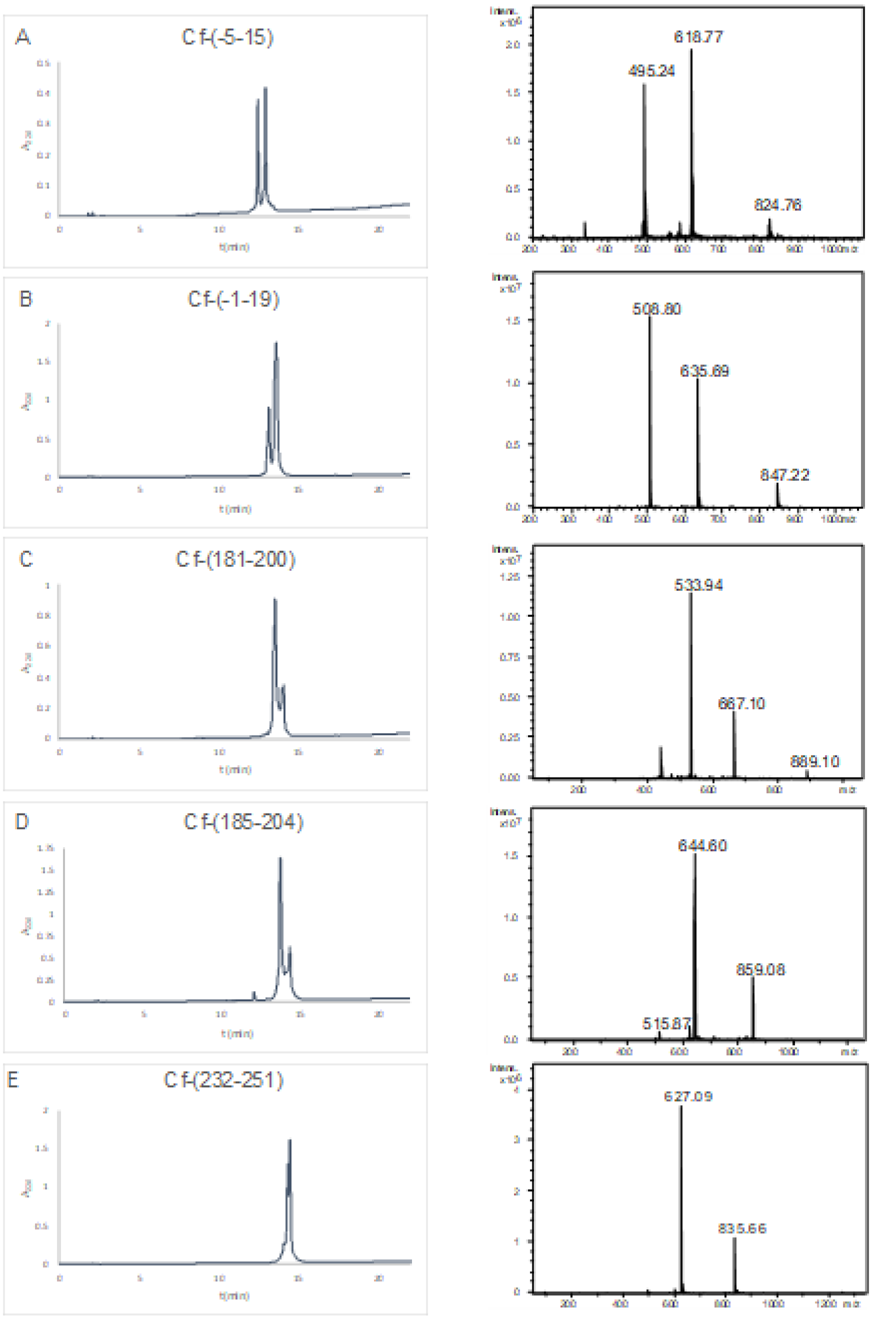

Figure S5. RP-HPLC chromatograms and ESI-MS spectra of Cf-HSV peptides prepared manually on Mimotopes lanterns. Double HPLC peaks often, but not always, occur, showing HPLC separation of 5- and 6-carboxyfluorescein labelled peptides resulting from the use of 5(6)-carboxyfluorescein. Exformma EX1600 analytical HPLC system, Waters Symmetry®, C18, 4.6x150 mm, 100 Å column; gradient: 0 B%, 0-2 min; 2-90 B%, 2-22 min; flow rate: 1 ml/min, detector wavelength: λ = 220 nm; A eluent: 0.1% TFA/water (V/V%), B eluent: 0.1% TFA/acetonitrile/water 80:20 (V/V%). ESI-MS was performed on Bruker Esquire 3000 Plus Ion Trap Mass Spectrometer (Bremen, Germany), continuous sample injection at a 10 µL/min flow rate. Samples were dissolved in 50% acetonitrile–50% water containing 0.1% acetic acid. Mass spectra were recorded in positive mode in the *m*/*z* 50–2000 range.

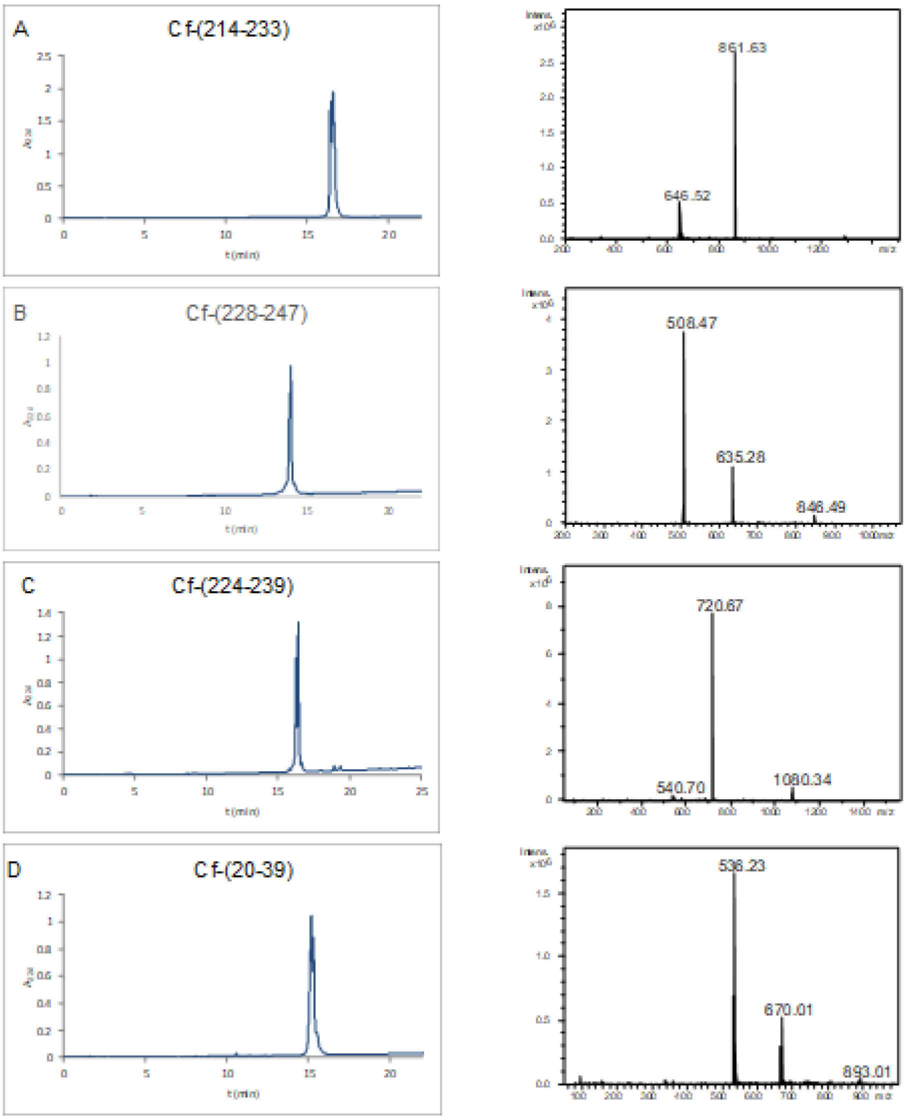

Figure S6: RP-HPLC chromatograms and ESI-MS spectra of Cf-HSV peptides prepared by Syro-I automated peptide synthesiser. Double HPLC peaks often, but not always, occur, showing HPLC separation of 5- and 6-carboxyfluorescein labelled peptides resulting from the use of 5(6)-carboxyfluorescein. Exformma EX1600 analytical HPLC system, Waters Symmetry®, C18, 4.6x150 mm, 100 Å column; gradient: 0 B%, 0-2 min; 2-90 B%, 2-22 min; flow rate: 1 ml/min, detector wavelength: λ = 220 nm; A eluent: 0.1% TFA/water (V/V%), B eluent: 0.1% TFA/acetonitrile/water 80:20 (V/V%). ESI-MS was performed on Bruker Esquire 3000 Plus Ion Trap Mass Spectrometer (Bremen, Germany), continuous sample injection at a 10 μL/min flow rate. Samples were dissolved in 50% acetonitrile–50% water containing 0.1% acetic acid. Mass spectra were recorded in positive mode in the *m*/*z* 50–2000 range.

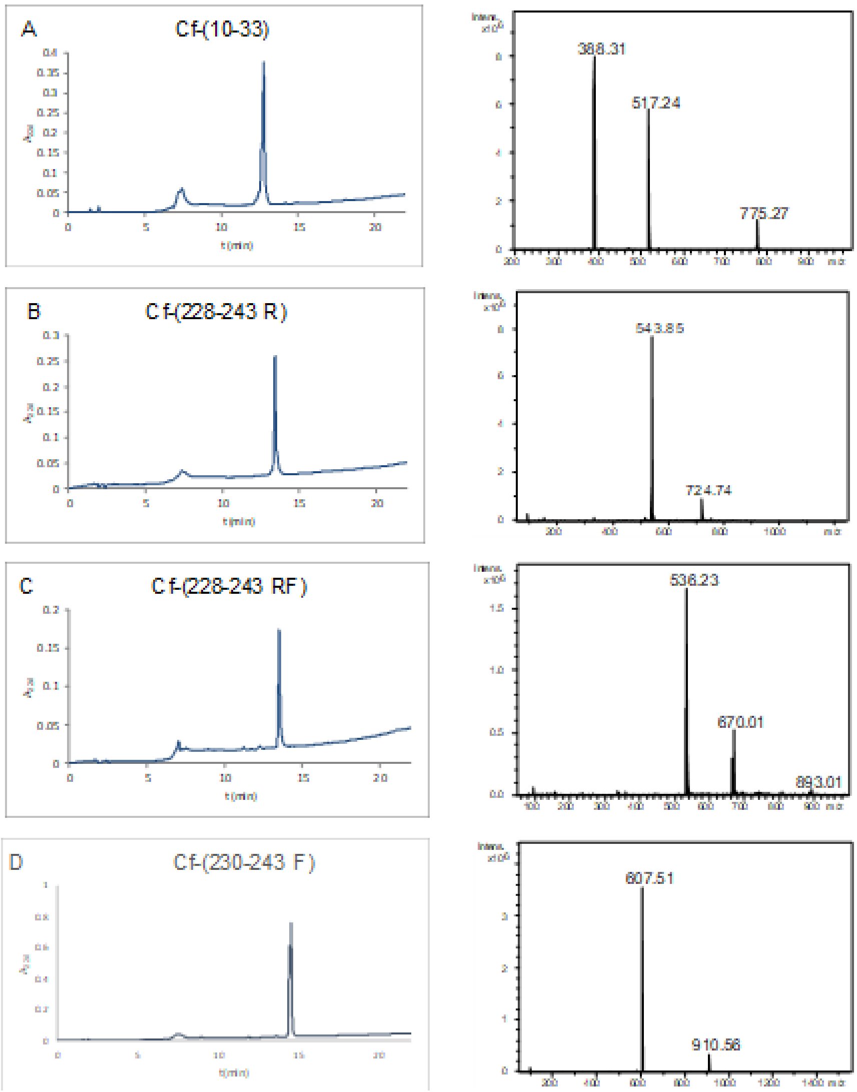

Figure S7. RP-HPLC chromatograms and ESI-MS spectra of Cf-HSV peptides prepared manually on Rink amide MBHA resin. Double HPLC peaks often, but not always, occur, showing HPLC separation of 5- and 6-carboxyfluorescein labelled peptides resulting from the use of 5(6)-carboxyfluorescein. Exformma EX1600 analytical HPLC system, Waters Symmetry®, C18, 4.6x150 mm, 100 Å column; gradient: 0 B%, 0-2 min; 2-90 B%, 2-22 min; flow rate: 1 ml/min, detector wavelength: λ = 220 nm; A eluent: 0.1% TFA/water (V/V%), B eluent: 0.1% TFA/acetonitrile/water 80:20 (V/V%). ESI-MS was performed on Bruker Esquire 3000 Plus Ion Trap Mass Spectrometer (Bremen, Germany), continuous sample injection at a 10 μL/min flow rate. Samples were dissolved in 50% acetonitrile–50% water containing 0.1% acetic acid. Mass spectra were recorded in positive mode in the m/z 50–2000 range.

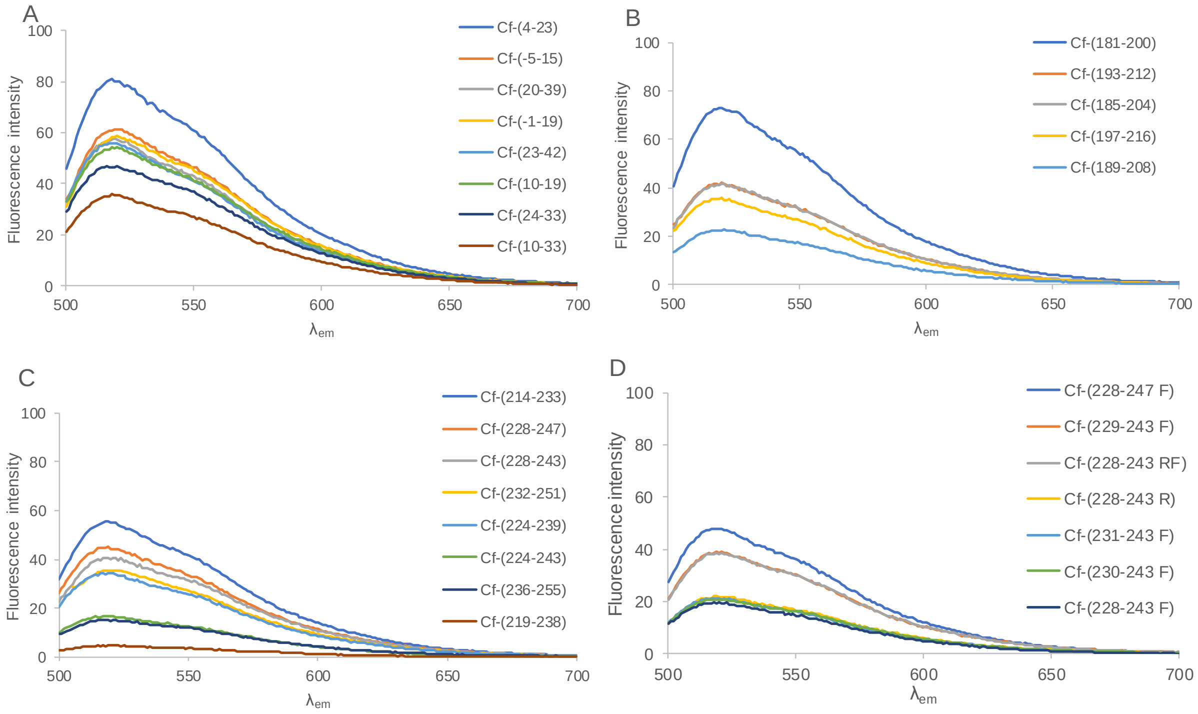

Figure S8. Fluorescence emission spectra of Cf-HSV-1 gD peptides at pH = 4.0. Peptides were dissolved in 0.1 M citrate phosphate buffer; c = 0.4 μM and measured with Varian Cary Eclipse fluorimeter. Excitation wavelength was λ = 488 nm. (**a**) Set I peptides, (**b**) Set II peptides, (**c**) Set III peptides, (**d**) Set IV peptides

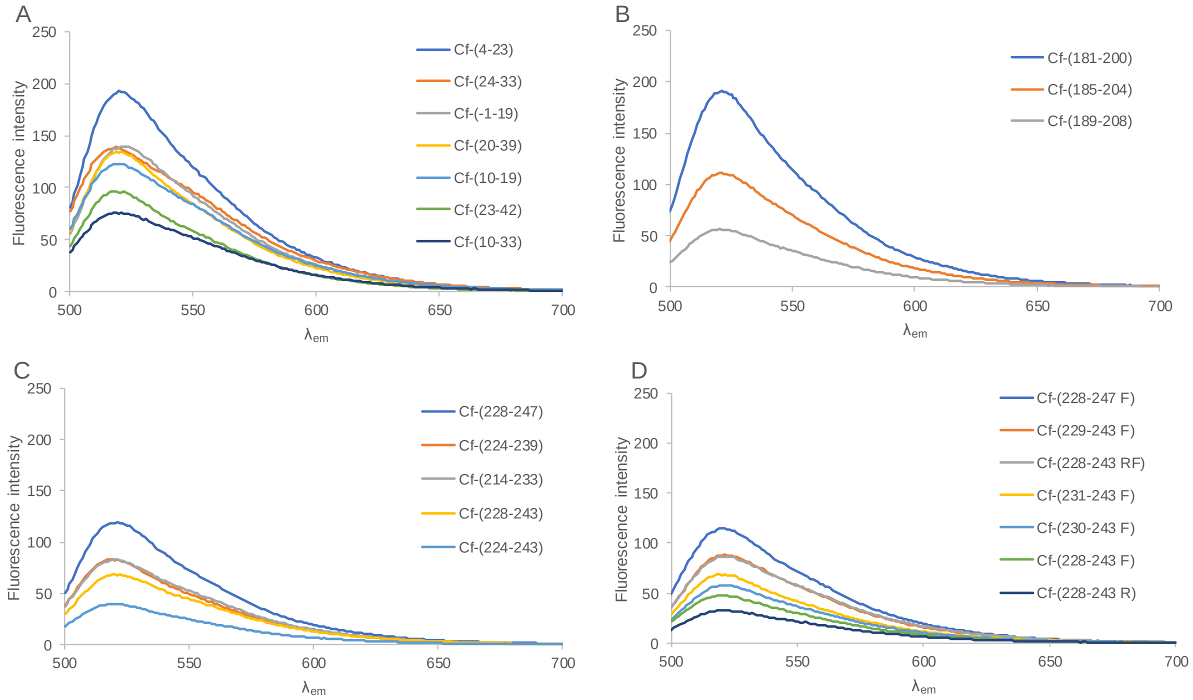

Figure S9. Fluorescence emission spectra of Cf-HSV-1 gD peptides at pH = 5.0. Peptides were dissolved in 0.1 M citrate phosphate buffer; c = 0.4 μM and measured with Varian Cary Eclipse

fluorimeter. Excitation wavelength was λ = 488 nm. (**a**) Set I peptides, (**b**) Set II peptides, (**c**) Set III peptides, (**d**) Set IV peptides

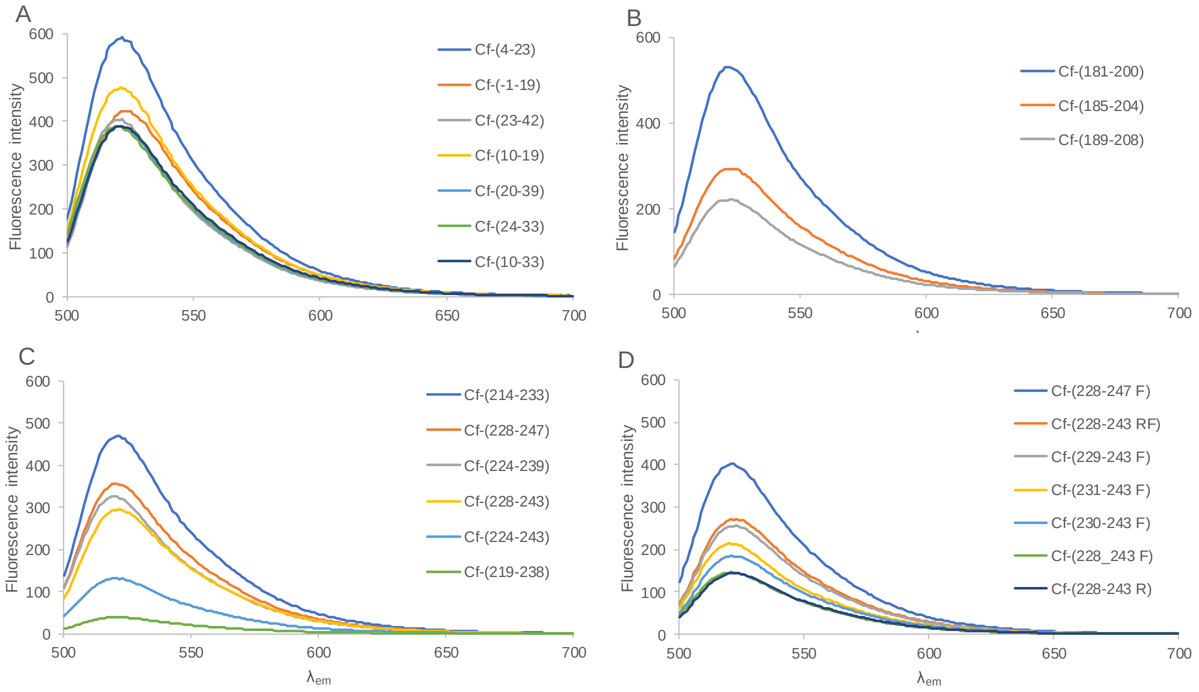

Figure S10. Fluorescence emission spectra of Cf-HSV-1 gD peptides at pH = 6.0. Peptides were dissolved in 0.1 M citrate phosphate buffer; c = 0.4 μM and measured with Varian Cary Eclipse fluorimeter. Excitation wavelength was *λ* = 488 nm. (**a**) Set I peptides, (**b**) Set II peptides, (**c**) Set III peptides, (**d**) Set IV peptides

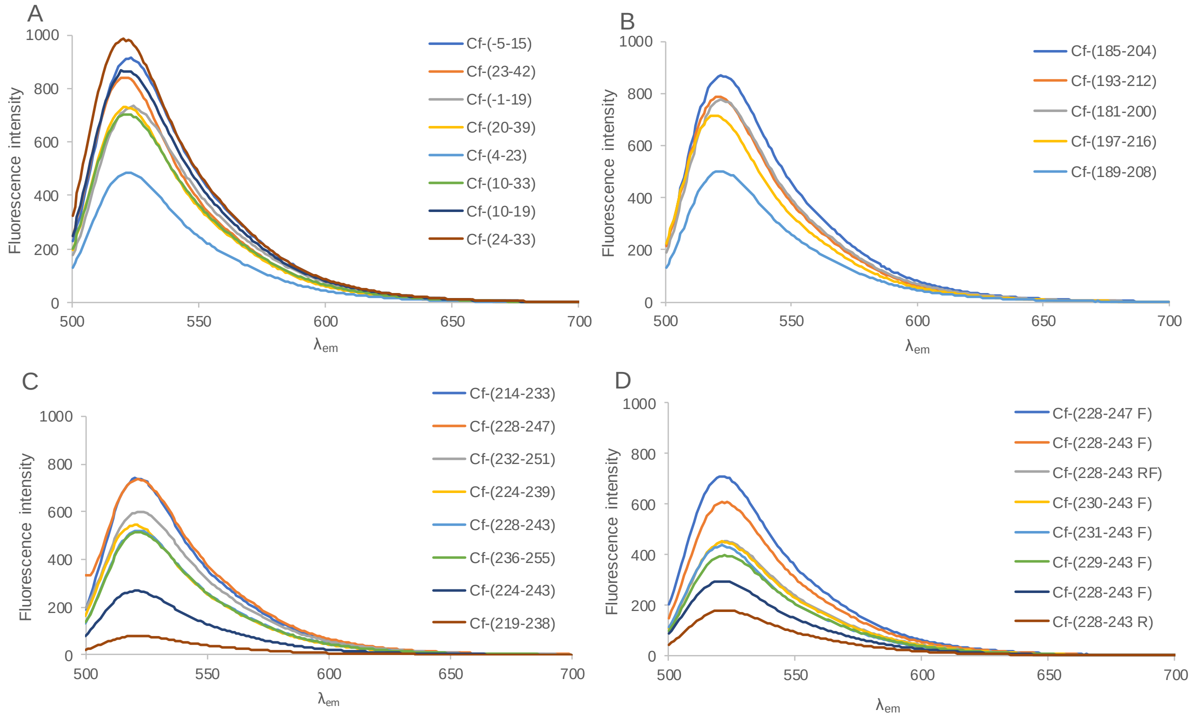

Figure S11. Fluorescence emission spectra of Cf-HSV-1 gD peptides at pH = 7.0. Peptides were dissolved in 0.1 M citrate phosphate buffer; c = 0.4 μM and measured with Varian Cary Eclipse

fluorimeter. Excitation wavelength was λ = 488 nm. (**a**) Set I peptides, (**b**) Set II peptides, (**c**) Set III peptides, (**d**) Set IV peptides

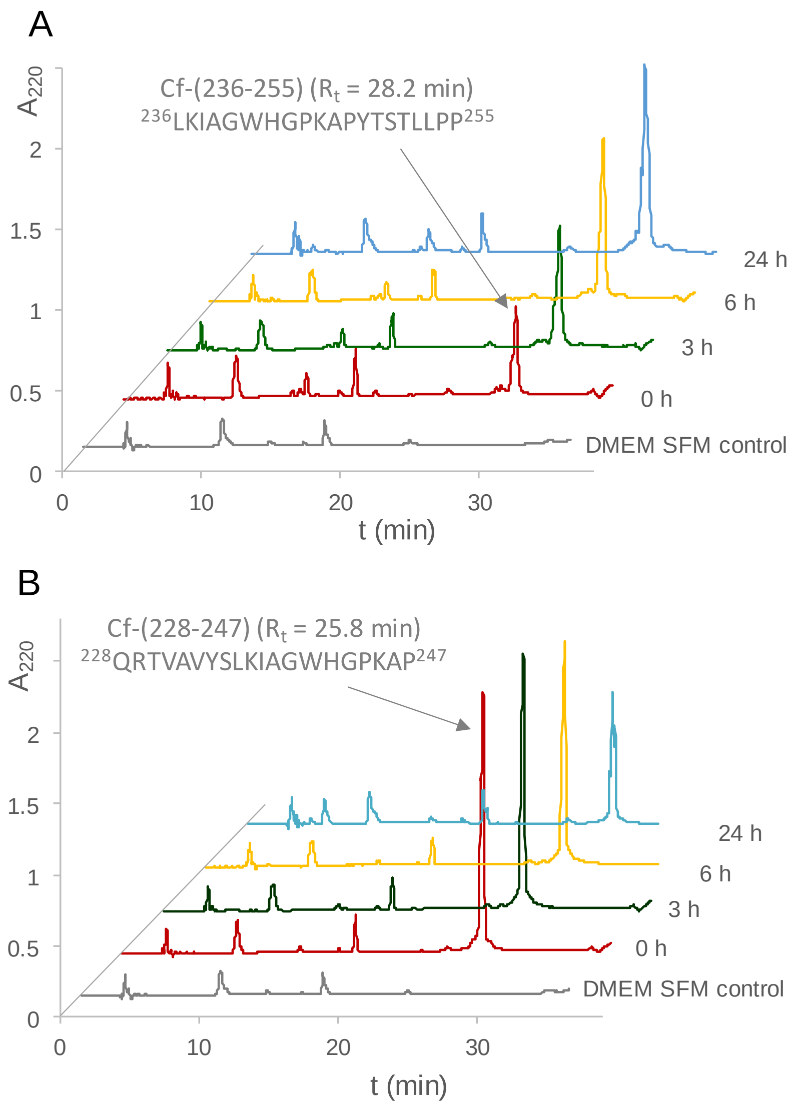

Figure S12. Stability study of (**a**) Cf-(236-255) and (**b**) Cf-(228-247), dissolved in DMEM serum free medium and incubated at 37 °C, followed by RP-HPLC. Samples for RP-HPLC (Knauer, Berlin, Germany) were taken at 0, 3, 6 and 24 hrs. Phenomenex Luna C18 column (5 μm, 300 Å, 4.6 mm × 250 mm) was applied with a gradient of 0-5 min 5% eluent B elevated to 55% B during 25 min, where eluent A: 0.1% TFA / water, eluent B: 0.1% TFA / acetonitrile–water 80:20 (v/v). Control: DMEM serum free medium. In case of Cf-(236-255) evaporation during the incubation caused increasing peptide peaks. Both peptides contain oxidation sensitive Trp residue. Cf-(236-255) was stable even after 24 h, the rising intensity of the peak representing the peptide is due to evaporation during the incubation and sampling. In case of Cf-(228-247) decreased intensity of the intact peptide was observed after 24 h. For the cellular uptake studies 3 h incubation is applied, and the peptides proved to be stable during this interval.

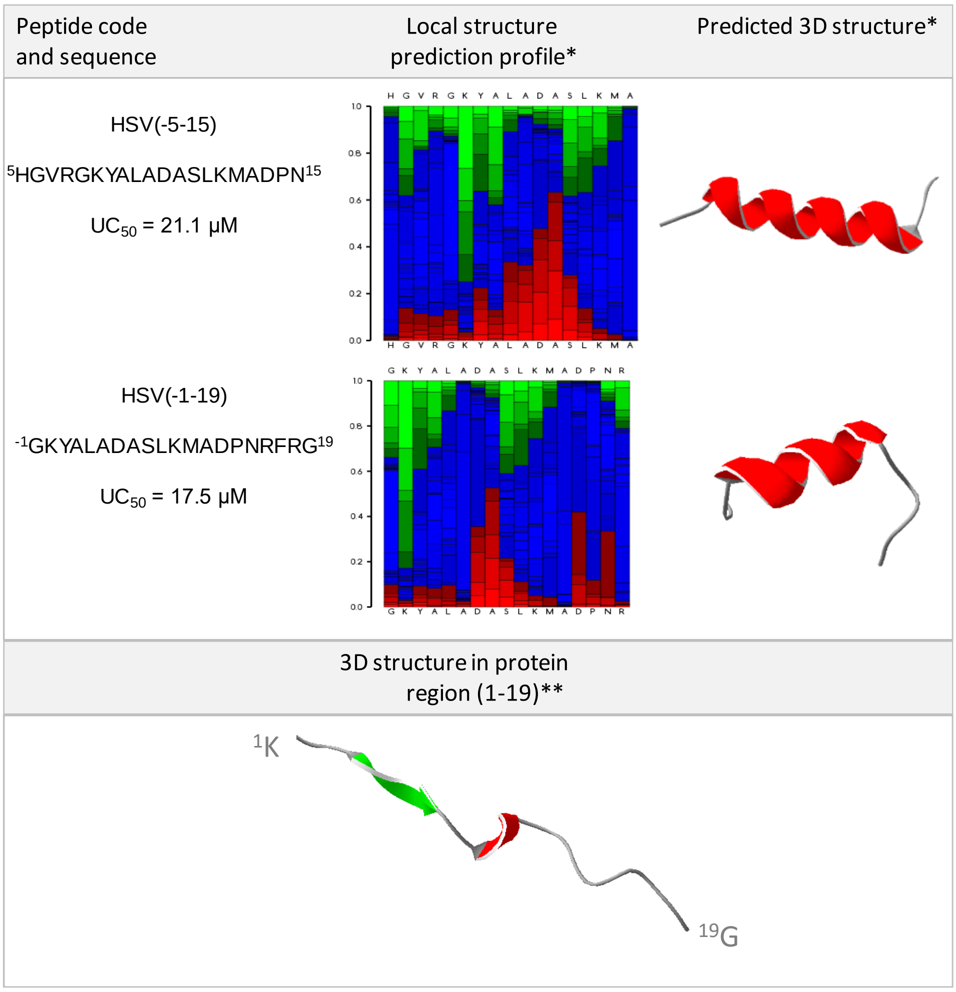

Figure S13. Upper panel: Predicted secondary structure of HSV-1 gD 20mer peptides of HSV-(-5–19) region using PEP-FOLD3 method. Lower panel: 3D structure of the HSV-(1–19) region within the HSV-1 gD protein (PDB ID: 1JMA)

* Graphical representation of the probabilities of Structural Alphabets (SA), corresponding to fragments of 4 residue length, at each position of the sequence. Colour code: red: helical, green: extended, blue: coil. The predicted structures of the region are displayed by the usual 3D ribbon structure.

** PBD ID: 1JMA

Note: Shen *et al.* [37]: "We emphasize that many structures were solved in aqueous solution with some buffers, containing 10–50 mM potassium phosphate (e.g., 2k76 and 2bkl) along with 100–200 mM NaCl (e.g., 1by0 and 1vpu) or even trifluoroethanol (TFE) (e.g., 2gdl and 2kya)." (NMR structure of 9-23mer, 25-50mer, various solvents).

Peptide code and sequence	Local structure prediction profile*	Predicted 3D structure*
HSV-(214-233)<br>^{214}VDSIGMLPRFIPENQRTVAV^{233}<br>UC_{50} = 26.5 μM	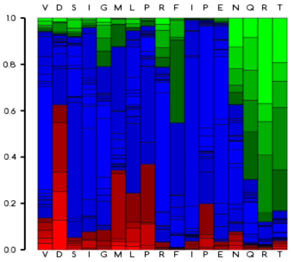	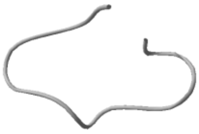
HSV-(219-238)<br>^{219}MLPRFIPENQRTVAVYSLKI^{238}<br>UC_{50} = 12.2 μM	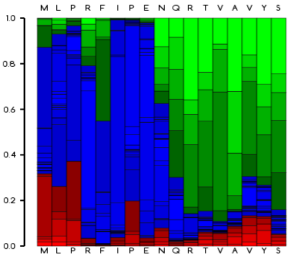	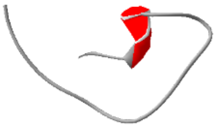
HSV-(224-243)<br>^{224}IPENQRTVAVYSLKIAGWHG^{243}<br>UC_{50} = 6.6 μM	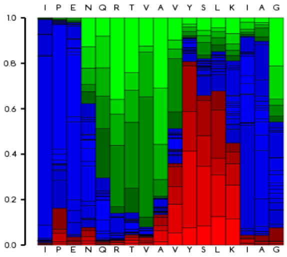	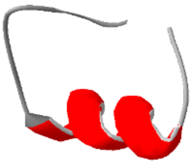

3D structure in protein region (214-243)**

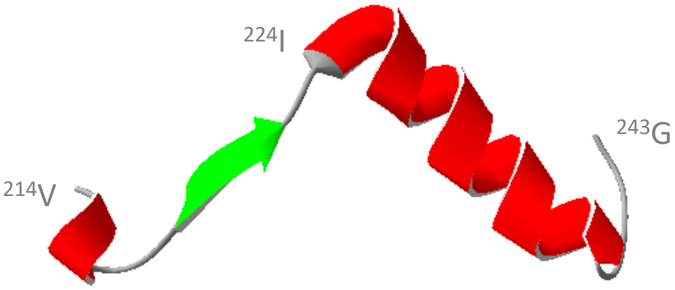

Figure S14. Upper panel: Predicted secondary structure of HSV-1 gD 20mer peptides of HSV-(214–243) region using PEP-FOLD3 method. Lower panel: 3D structure of the HSV-(214–243) region within the HSV-1 gD protein (PDB ID: 1JMA)

* Graphical representation of the probabilities of Structural Alphabets (SA), corresponding to fragments of 4 residue length, at each position of the sequence. Colour code: red: helical, green: extended, blue: coil. The predicted structures of the region are displayed by the usual 3D ribbon structure.

** PBD ID: 1JMA

Peptide code and sequence	Local structure prediction profile*	Predicted 3D structure*
HSV-(228-247)<br>^{228}QRTVAVYSLKIAGWHGPKAP^{247}<br>UC_{50} = 4.7 µM	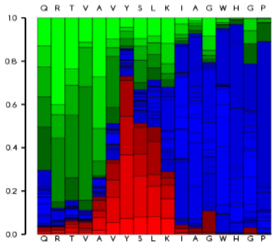	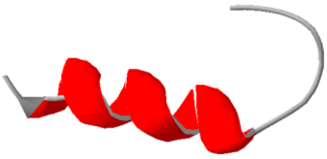
HSV-(232-251)<br>^{232}AVYSLKIAGWHGPKAPYTST^{251}<br>UC_{50} = 18.0 µM	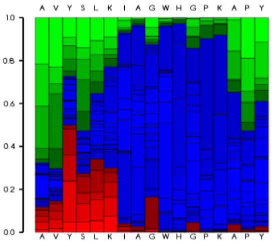	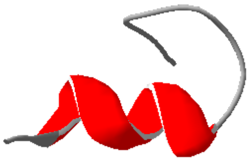
HSV-(236-255)<br>^{236}LKIAGWHGPKAPYTSTLLPP^{255}<br>UC_{50} = 65.7 µM	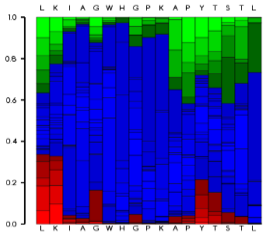	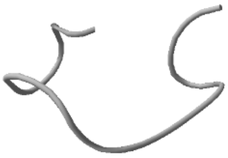

3D structure in protein region (228-255)**

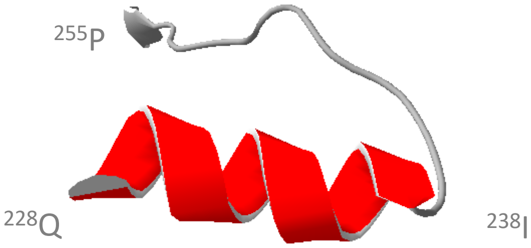

Figure S15. Upper panel: Predicted secondary structure of HSV-1 gD 20mer peptides of HSV-(228–255) region using PEP-FOLD3 method. Lower panel: 3D structure of the HSV-(228–255) region within the HSV-1 gD protein (PDB ID: 1JMA)

* Graphical representation of the probabilities of Structural Alphabets (SA), corresponding to fragments of 4 residue length, at each position of the sequence. Colour code: red: helical, green: extended, blue: coil. The predicted structures of the region are displayed by the usual 3D ribbon structure.

** PBD ID: 1JMA

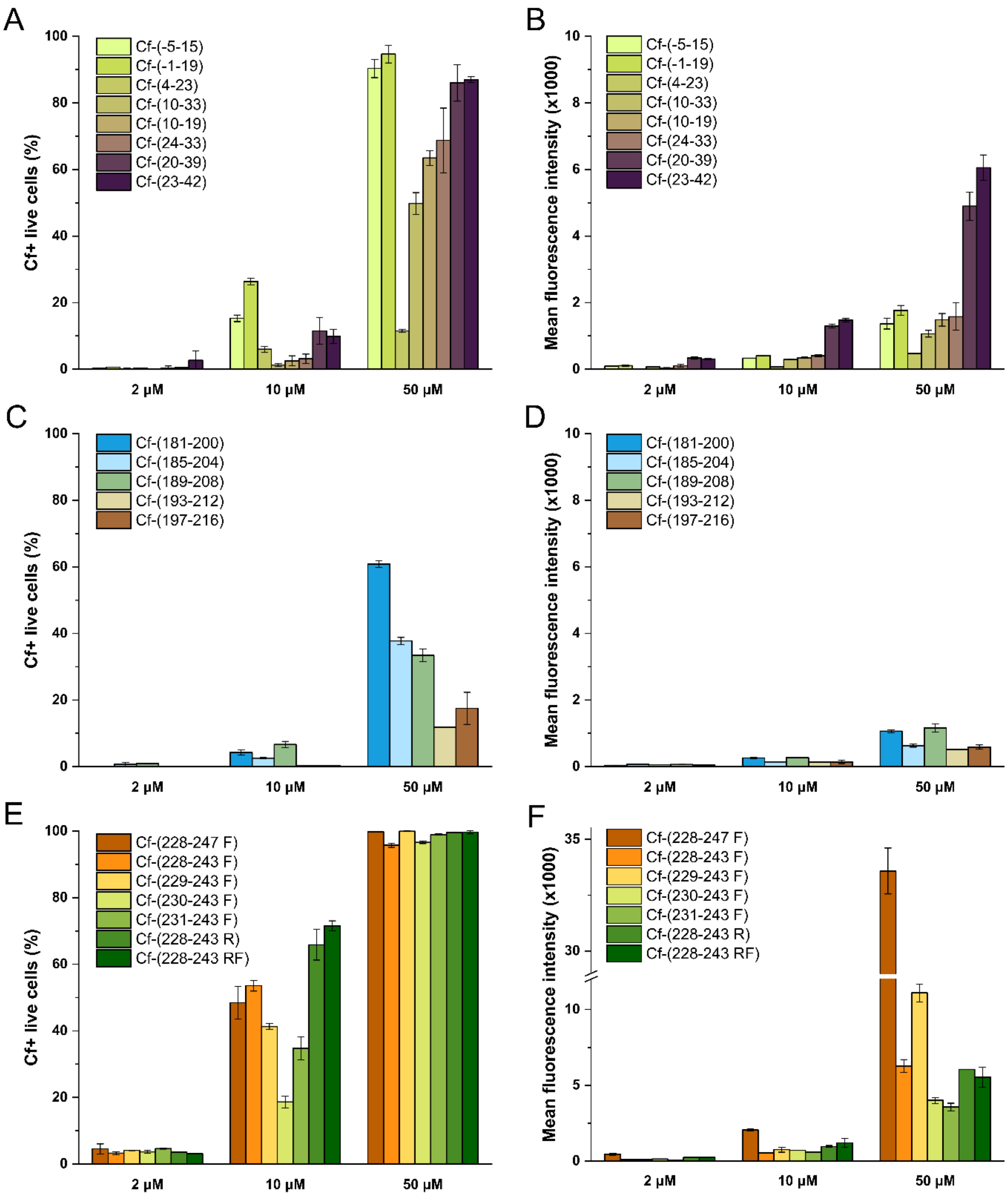

Figure S16. Internalisation of synthetic Cf-HSV-1 gD peptides at 2-50 μM concentration range into SH-SY5Y neuroblastoma cells measured by flow cytometry. (**a,b**) Set I peptides, (**c,d**) Set II peptides, (**e,f**) Set IV peptides. Graphs were plotted using Origin 2018 (OriginLab, Northampton, Massachusetts, USA)

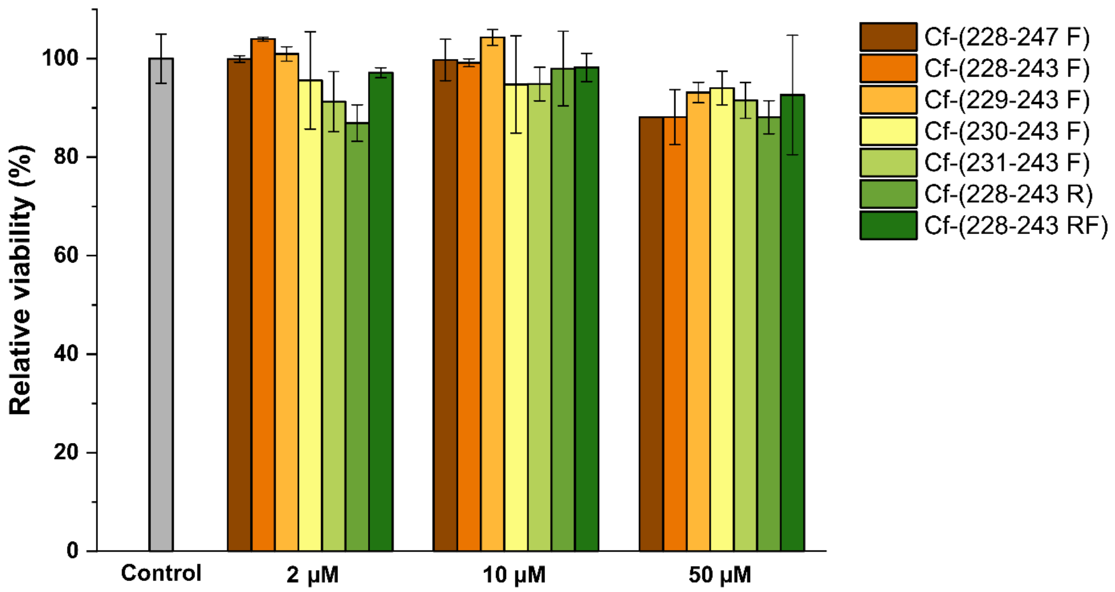

Figure S17. Relative viability of SH-SY5Y cells after 3 h treatment with Set IV Cf-HSV peptides, compared to untreated control. Graphs were plotted using Origin 2018 (OriginLab, Northampton, Massachusetts, USA)

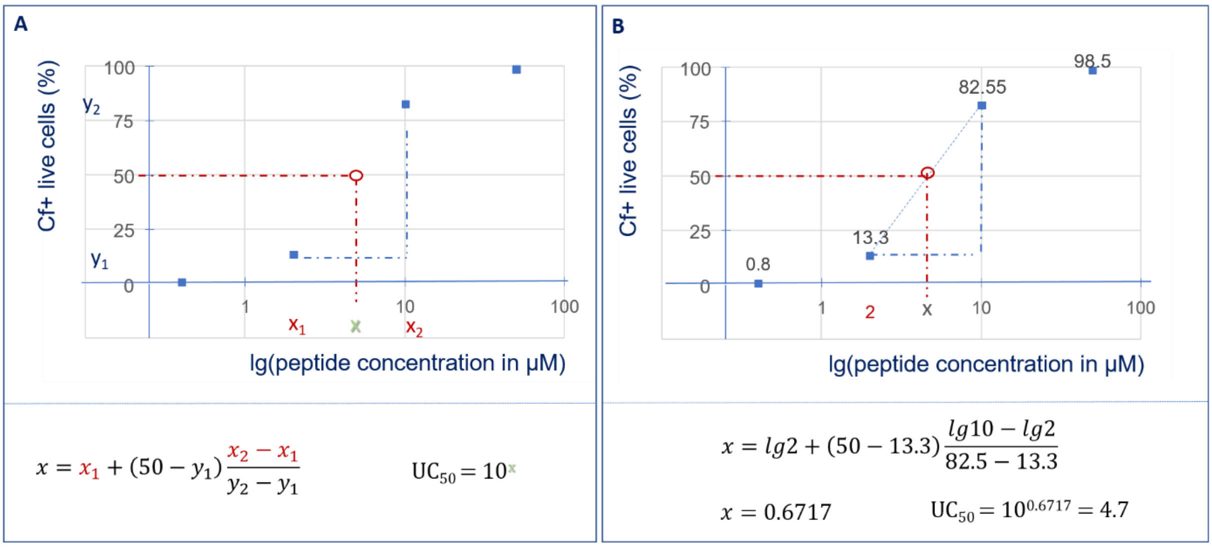

Figure S18. Calculation of UC_{50} values with Microsoft Excel. (**a**) General formula of linear interpolation of the logarithmic concentration x necessary for 50 % of the live cells showing intracellular fluorescence. x_1, x_2: logarithm of peptide concentration (in µM) under and above the expected x value, y_1 and y_2: Cf+ live cell percentage belonging to x_1 and x_2, respectively. (**b**) Formula applied to calculate the UC_{50} value of Cf-(228-247) peptide. UC_{25} or UC_{10} values corresponding to 25 or 10 % of cells having intracellular fluorescence can be determined similarly.

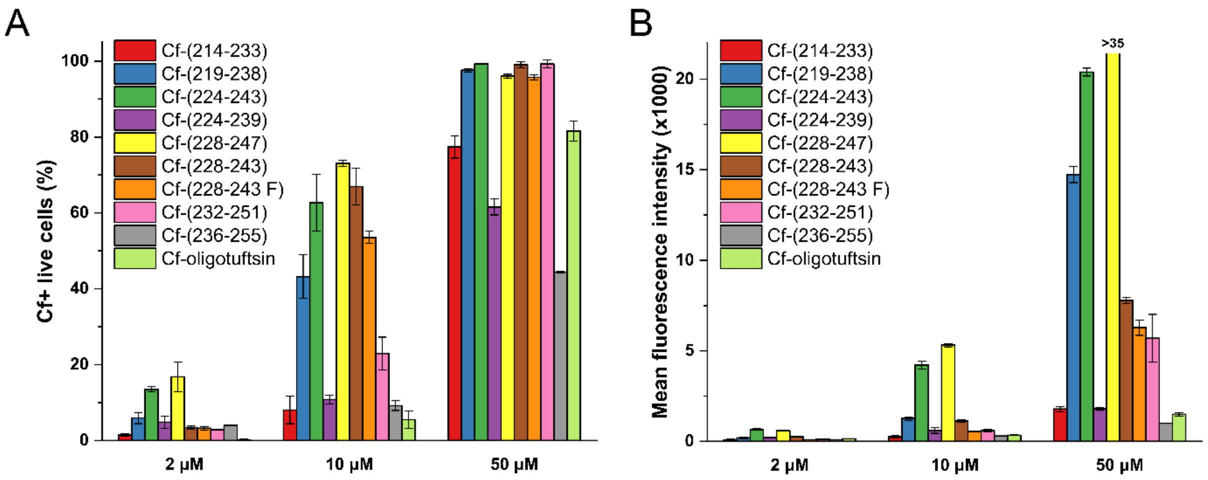

Figure S19. Comparison of the internalisation of Set III and Cf-(228-243 F) Cf-HSV-1 gD peptides with oligotuftsin Cf-OT10 (Cf-TKPKGTKPKG) into SH-SY5Y neuroblastoma cells measured by flow cytometry. Data of Cf-HSV peptides can also be seen in Figure 6a,b of the main text. Graphs were plotted using Origin 2018 (OriginLab, Northampton, Massachusetts, USA)

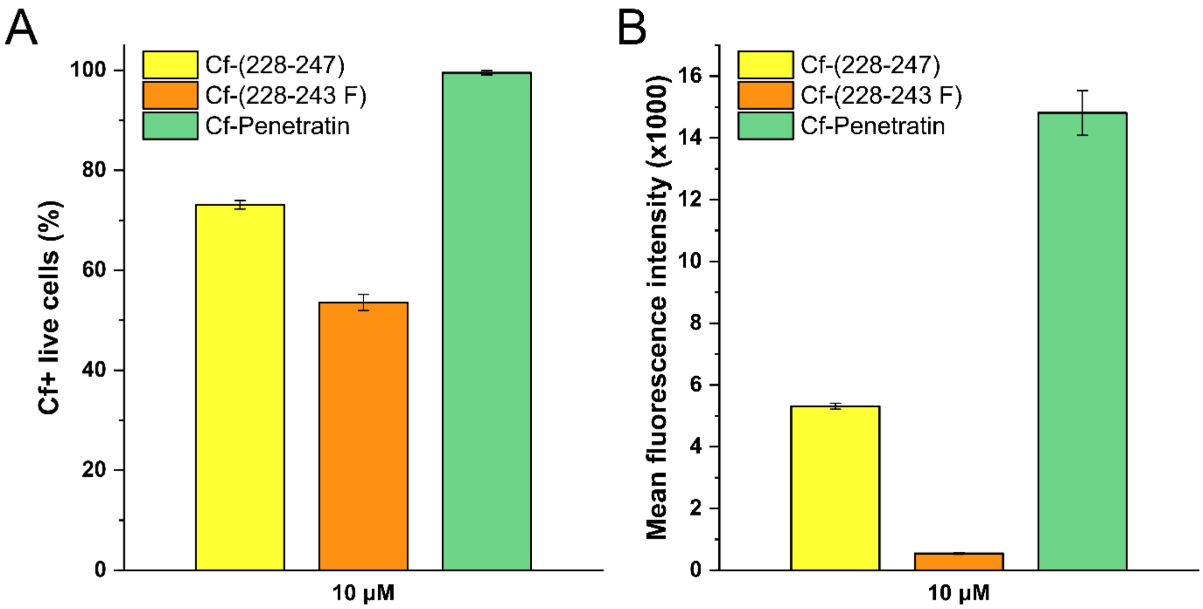

Figure S20. Comparison of the internalisation of Cf-(228–247 and Cf-(228–243 F) peptides with Cf-Penetratin into SH-SY5Y neuroblastoma cells measured by flow cytometry. Graphs were plotted using Origin 2018 (OriginLab, Northampton, Massachusetts, USA)
